# The genetics and evolution of moth melanism in the absence of strong natural selection

**DOI:** 10.1093/nsr/nwaf441

**Published:** 2025-10-23

**Authors:** Yongjian Liu, Yunjie Pan, Yanghui Cao, Zhibo Wang, Chaomei Ge, Guiyun Li, Xiaojing Liu, Gangqi Fang, Yaohui Wang, Qiang Xiao, Shuai Zhan

**Affiliations:** State Key Laboratory of Plant Trait Design, CAS Center for Excellence in Molecular Plant Sciences, Chinese Academy of Sciences, Shanghai 200032, China; University of Chinese Academy of Sciences, Beijing 100049, China; State Key Laboratory of Plant Trait Design, CAS Center for Excellence in Molecular Plant Sciences, Chinese Academy of Sciences, Shanghai 200032, China; University of Chinese Academy of Sciences, Beijing 100049, China; Key Laboratory of Plant Protection Resources and Pest Management of the Ministry of Education, Entomological Museum, Northwest A&F University, Yangling 712100, China; Key Laboratory of Tea Quality and Safety Control, Tea Research Institute, Ministry of Agriculture, Chinese Academy of Agricultural Sciences, Hangzhou 310008, China; Key Laboratory of Tea Quality and Safety Control, Tea Research Institute, Ministry of Agriculture, Chinese Academy of Agricultural Sciences, Hangzhou 310008, China; State Key Laboratory of Plant Trait Design, CAS Center for Excellence in Molecular Plant Sciences, Chinese Academy of Sciences, Shanghai 200032, China; Jiangsu Key Laboratory of Sericultural Biology and Biotechnology, School of Biotechnology, Jiangsu University of Science and Technology, Zhenjiang 212100, China; State Key Laboratory of Plant Trait Design, CAS Center for Excellence in Molecular Plant Sciences, Chinese Academy of Sciences, Shanghai 200032, China; Key Laboratory of Biology and Sustainable Management of Plant Diseases and Pests of Anhui Higher Education Institutes, College of Plant Protection, Anhui Agricultural University, Hefei 230036, China; Key Laboratory of Tea Quality and Safety Control, Tea Research Institute, Ministry of Agriculture, Chinese Academy of Agricultural Sciences, Hangzhou 310008, China; State Key Laboratory of Plant Trait Design, CAS Center for Excellence in Molecular Plant Sciences, Chinese Academy of Sciences, Shanghai 200032, China

**Keywords:** melanism, population genetics, Lepidoptera, parallel evolution, evolutionary hotspot

## Abstract

Melanism is a common phenotypic variation in wild animals, and it provides valuable models for understanding how natural selection shapes biodiversity. The tea geometrid is prevailing in tea gardens of Asia and exhibits natural polymorphism in body and wing colors. Based on genetic linkage analysis, we mapped the melanism locus of this species to a 500 kb genomic interval, the ‘*cortex* locus’ that controls industrial melanism in distantly related moths in Britain. Investigations of different natural populations of tea geometrids and related species reveal the presence of multiple independent melanic loci within this highly variable hotspot region. Functional studies suggest that the major effector within this locus is the conserved microRNA, *mir-193*, which produces melanin pigment during pupal stages. By examining the evolutionary and ecological contexts of melanism in tea geometrids, we find that, in the apparent absence of strong natural selection such as camouflage advantage, potential reproductive disadvantages might contribute to the low genetic diversity and the minor fraction of melanic morphs in wild populations. Our study synthesizes genetic and ecological information to propose an evolutionary scenario in which a highly variable hotspot locus may fuel the repeated occurrence of non-adaptive phenotypic variations, ultimately shaping the biodiversity in nature.

## INTRODUCTION

Phenotypic variations are common within and between animal species and have been found to occur repeatedly in many lineages. The occurrence of these polymorphisms often serves as material to study how natural selection drives trait evolution and speciation. Melanism in moths is well known owing to the textbook example of industrial melanism in British peppered moths, *Biston betularia* (Lepidoptera: Geometridae) [1,2]. During the Industrial Revolution, the common pale form (*typica*) of *B. betularia* in Britain was rapidly replaced by a black form (*carbonaria*) due to the advantage of camouflage on polluted surfaces [3–6]. This phenomenon greatly agrees with Darwin’s theory of ‘survival of the fittest’. In addition, there are other mechanisms underlying melanism in nature, including non-visual factors (e.g. thermoregulation and UV resistance) and pleiotropic effects by other associated traits (e.g. immunity and phase changes) [7–9]. However, the evolution of melanism under diverse selection scenarios remains largely unknown.

Elucidating the genetic bases of melanism could advance our understanding of its ecological consequences. In British peppered moths, industrial melanism has been associated with mutations within the *cortex* locus [5], a hotspot region that also controls melanism in other geometrid moths, as well as wing color patterns in some butterflies [10,11]. Unlike the extensive research conducted on butterflies, which have identified a handful of loci controlling wing color patterns across different lineages [5,11–14], studies with similar resolution have not been pursued on moths that comprise 90% of the described Lepidoptera (moths and butterflies). Particularly, it remains uncertain whether there are additional loci responsible for melanism or other color variations in moths in nature.

**Figure 1.**
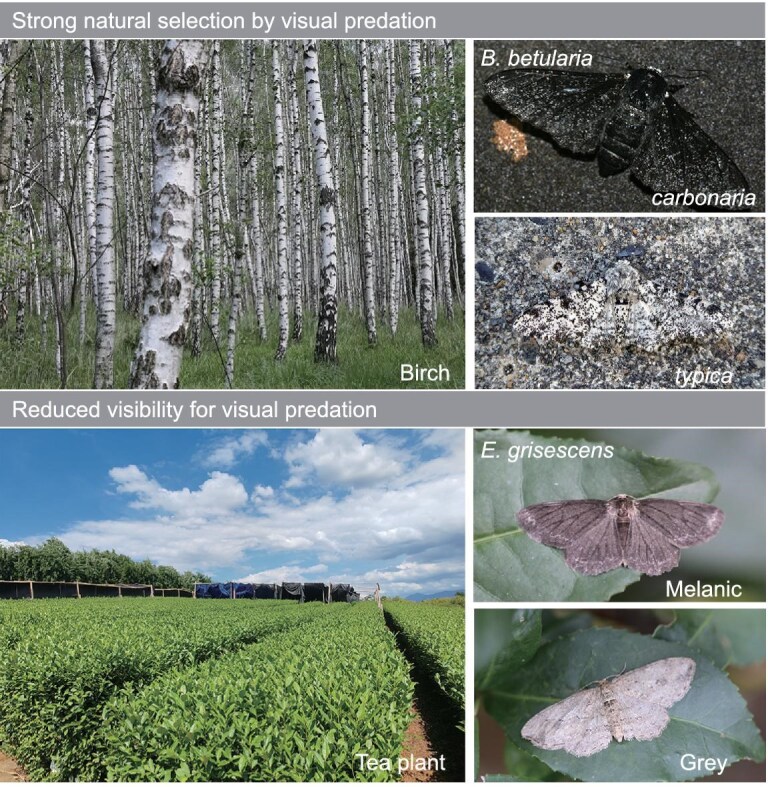
Different ecological contexts of melanism in moths. Top: marked visual camouflage advantage of body color variations in the peppered moth (*B. betularia*) in its ecological context. In brief, the pale morph (*typica*) is well camouflaged on the lichen-covered barks, while the melanic morph (*carbonaria*) is camouflaged against a dark background (e.g. polluted tree barks). Bottom: the major ecological context of the tea geometrid (*E. grisescens*), since its adaptation to tea plants. In tea gardens (represented bottom left), tea plants grow as dense branched shrubs, making visual recognition from aerial views more difficult. In addition, both color morphs (melanic and gray) are not well camouflaged against their resting backgrounds of leaves or twigs. Thus, strong selection by visual predators seems to be absent or unclear for the color variations in tea geometrids. On the other hand, the gray morph exhibits a common texturing and coloration that is widespread among moths across various visual backgrounds. It is likely that the lack of strong selection is more evident in the melanic morph. Photo credit: Silver birch (*Betula pendula*) trees, by T. Kebert on Wikimedia Commons CC BY-SA 4.0; peppered moth pale morph (*typica*), by AJC1 on Wikimedia Commons CC BY-SA 2.0; peppered moth melanic morph (*carbonaria*), by Ben Sale on Wikimedia Commons CC BY-SA 2.0.

The tea geometrid (*Ectropis grisescens*, Warren 1894) is a species of geometrid moth that is widely distributed across East Asia (from 24°N to 32°N). As a primary leaf-chewing pest on cultivated tea plants, this species occurs predominantly in the major tea-producing regions of China. Natural populations of tea geometrids exhibit black and gray forms of adults (Fig. 1), phenocopying the polymorphism of the peppered moth and other geometrids associated with industrial melanism in Britain (e.g. *Phigalia pilosaria*, *Odontopera bidentata*, *Paradarisa consonaria* and two species from the genus *Ectropis*) [15]. Selective predation by insectivorous birds is a key factor driving changes in the frequency of melanism in British peppered moths, in which black morphs are better camouflaged against dark backgrounds, i.e. tree barks (birch or willow) darkened by coal pollution [16] (Fig. 1). However, such strong natural selection appears to be absent or unclear in the ecological context of tea geometrids. In tea gardens, tea plants typically grow as densely branched shrubs, whose trunks are fully sheltered by leaves and twigs from aerial views (Fig. 1). This scenario further minimizes the advantage of body color variations for crypsis, if at all. Thus, the color variation in tea geometrids offers a unique opportunity to trace the parallel evolution of melanism under different geographical, ecological and selective scenarios. Here, we utilized canonical linkage mapping and population genomic approaches to investigate the genetic basis of melanization in this system, which would enable us to compare the parallel evolutionary paths across geographically and genetically distant species. Moreover, we assessed the frequencies of melanism in the field and associated them with ecological factors to infer the selective forces that have driven its evolution.

## RESULTS

### Characterization of the melanism locus in tea geometrid moths

**Figure 2.**
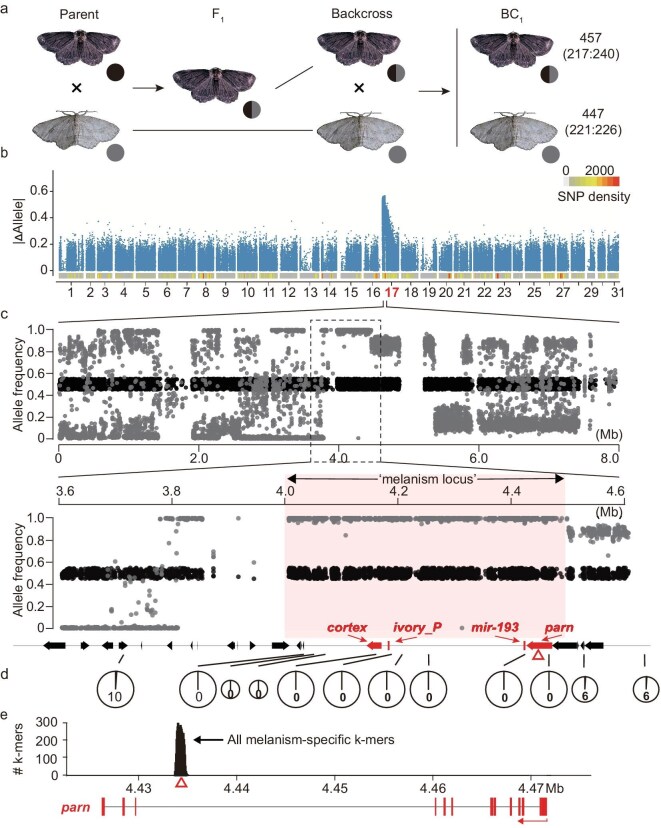
Characterization of the melanism locus of tea geometrids based on cross-mapping. (a) Schematic diagram of a backcross mapping population (BC_1_) from gray and melanic morphs of the tea geometrid. In brief, a melanic adult and a gray adult were crossed to produce F_1_ hybrid offspring, which were further backcrossed with the gray morph (recessive parent) to produce the BC_1_ population. The numbers indicate the individuals of each morph (female:male). (b) Genome-wide plot of allele difference between gray and melanic morphs. Each dot indicates the absolute value of the allele difference between the pooled gray and melanic offspring of BC_1_. The bar plot at the bottom illustrates SNP density along with each chromosome. (c) The enlarged view of the subregions with top divergence signatures between melanic and gray morphs. Gray and black dots indicate the derived allele frequency within the gray and melanic offspring of BC_1_, relative to the allele of a melanic reference genome. The encompassed protein-coding genes, together with *mir-193* and conserved ivory promotor (named as *ivory_P*) are indicted as below. All focal locus is highlighted in red. (d) Identification of cross events around the candidate locus based on PCR of the BC_1_ progeny. The lines of each pie chart link to the genomic position of the developed markers. The size of pie charts represents the total number of individuals being investigated (*n* = 208–898), while the number indicates the detected cross events. (e) Distribution of k-mers unique to the melanic offspring. Histograms indicate the mapped reads containing the k-mers that are present in all melanic BC_1_ offspring but absent in all gray ones. The peak is located at the internal intron of *parn*.

We applied linkage mapping to explore the genetics of the melanism phenotype in tea geometrids. Both the forward and reciprocal crosses between a pair of melanic and gray parents produced F_1_ offspring that consistently displayed the melanism phenotype, with an approximately 1:1 sex ratio (Fig. 2a; Table S1). Self-crossing among F_1_ individuals produced an F_2_ population that exhibits trait segregation, with an approximate 3:1 ratio between melanic and gray forms (Table S1). We also backcrossed the F_1_ offspring with the gray parent, leading to an approximately 1:1 ratio between melanic and gray in the resulting BC_1_ population (Tables S1 and S2). These segregation patterns jointly reveal that melanism in tea geometrids is a dominant trait, likely controlled by a single Mendelian locus on an autosome.

We initially used a candidate gene approach by investigating the expression profiles of seven key genes involved in the canonical melanin synthesis pathway in the wing tissues of various developmental stages (Fig. S1). However, no significant difference was observed in the expression of these genes between melanic and gray individuals. The only exception was *yellow*, which showed an unexpected increase during the late pupal stage in gray forms, contrary to its expected role in the pathway (Fig. S1). The variants within the coding regions of these genes were also genotyped by polymerase chain reaction (PCR) in pooled samples of melanic and gray segregants (see Materials and Methods), but none showed a significant association with melanization (Table S3).

To characterize the causal locus directly, we produced a BC_1_ crossing progeny consisting of 904 individuals, which included 457 melanic and 447 gray ones, for sequencing-based bulked segregant analysis (Fig. 2a; Table S4). A total of 767 Gb of sequencing data (∼1000× coverage of *E. grisescens* genome) was generated for scoring genotypes at ∼268 000 effective single nucleotide polymorphisms (SNPs) (see Materials and Methods). It can be anticipated that the majority of SNPs will segregate in nearly equal ratios between the melanic and gray samples in the BC_1_ progeny, while the causal locus is expected to have an enrichment of SNPs with marked differences in allele frequency between the gray pool (fixed) and the melanic pool (∼0.5). Remarkably, we found such signatures exclusively within a candidate segment on chromosome 17 (Fig. 2b), ranging from 3.8 to 4.5 Mb. In this segment, gray individuals exhibited fixed alleles, while melanic individuals showed heterozygous alleles approaching a ratio of 0.5 (Fig. 2c). We further developed molecular markers within this candidate region and independently scored the BC_1_ progeny using PCR. The genotyping results showed that there were no further crossing events within the subregion of 4.0 to 4.5 Mb (Fig. 2d; Table S5). In addition, we characterized a strong linkage disequilibrium (LD) block that exactly spans this locus, indicative of a tightly linked signature (Fig. S2). This 500 kb non-recombinant region encompasses seven annotated protein-coding genes, including *cortex* that was identified as the contributor to industrial melanism in British peppered moths [5]. This interval is termed the ‘melanism locus’ hereafter (Fig. 2c).

On the other hand, it is noteworthy that partial regions within the candidate locus lack effective markers (Fig. 2c). *De novo* assembly of these focal regions, using long-read sequencing data, showed massive structural variations (SVs) between the gray and melanic morphs (Fig. S2). Since large SVs cannot be effectively identified by the mapping approach, we applied a k-mer-based approach to directly analyze the presence and absence of DNA segments from the sequencing reads (see Materials and Methods). We identified 463 unique 31-mers that were present in all melanic BC_1_ individuals but completely absent in gray samples. Notably, all these 31-mers were located at a unique locus at the third intron of the gene *parn*, which encodes poly(A)-specific ribonuclease (PARN) and is located 260 kb upstream of *cortex* (Fig. 2e).

### Independent origins of melanism in different tea geometrid populations

**Figure 3.**
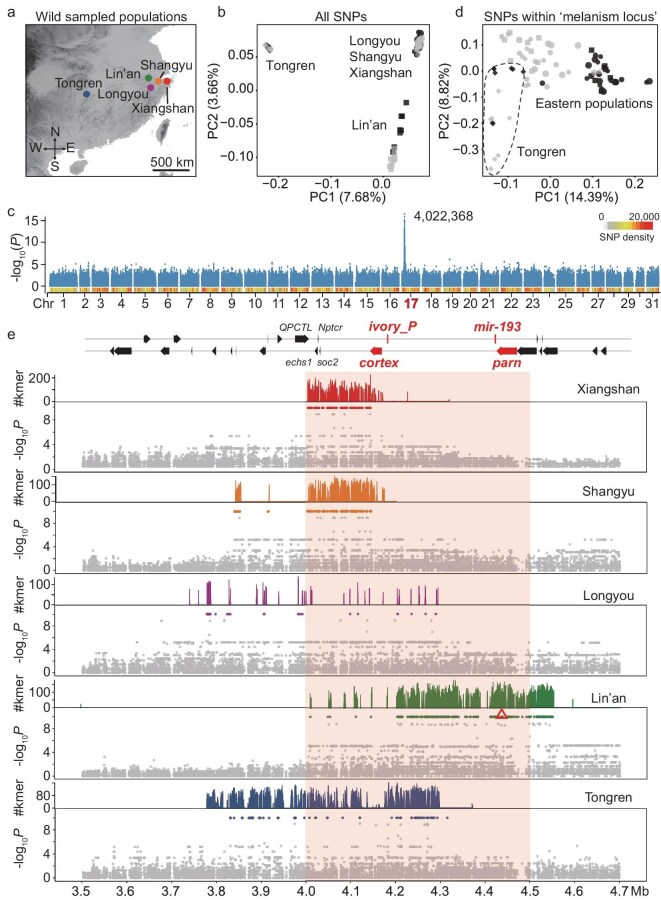
Characterization of the melanism locus of tea geometrids in wild populations. (a) Sampling sites of wild tea geometrid populations for comparing the genomes of gray and melanic morphs. (b) PCA of sequenced individuals based on genome-wide SNPs. Symbols in gray and black represent gray and melanic individuals, respectively, of various populations. (c) GWAS of the body color phenotype (gray or melanic) across all five sampled populations. The labeled number indicates the genomic position exhibiting the most significant *P* value. Bar plots at the bottom indicate SNP density. (d) PCA of involved individuals based on SNPs from the ‘melanism locus’ identified in Fig. 2c. The area outlined by dotted lines indicates the TR population. (e) GWAS on each wild population between gray and melanic morphs. For each population, the count of morph-specific k-mers, SNPs with fixed divergent alleles between the morphs, and the GWAS significance were plotted from top to bottom. The red triangle corresponds to those in Fig. 2c and e, i.e. the locus where melanic-specific k-mers of cross-mapping are located. Review drawing number: GS 京 (2025) 2216号.

We sampled wild populations from geographically distant tea-producing regions to test whether this mapped locus from a single cross-mapping progeny is commonly responsible for melanization in tea geometrids (Fig. 3a). A total of 40 melanic and 40 gray adults from five populations that exhibited both color forms were subjected to independent genome sequencing and genotyping, with 8 individuals of each morph per population (Table S6). Both principal component analysis (PCA) and ancestry estimation based on genome-wide SNPs clustered these samples into three main genetic groups, largely agreeing with their geographical locations, i.e. the western population from Tongren (TR) was distinctly separated from the eastern populations from Lin’an (LA), Xiangshan (XS), Shangyu (SY) and Longyou (LY) (Fig. 3b; Fig. S3).

Genome-wide association analysis (GWAS) involving samples from all populations identified a single outlier peak (*P *= 2.02 × 10^–17^), which exactly spans the ‘melanism locus’ identified by linkage mapping (Fig. 3c). When only the SNPs within the ‘melanism locus’ were being considered, melanic and gray individuals from eastern populations were clearly separated by the first PC, while the two morphs of the TR population were separated based on the second PC (Fig. 3d). These patterns support a common role of the ‘melanism locus’, but they also show genetic disparity of the melanism loci among populations. Indeed, we found that the top GWAS signatures correspond to diverse genomic intervals within this locus when geographical populations were analyzed independently. Only the LA population was enriched in association signatures and morph-specific k-mers around *parn*, with extension to the 5′ upstream of *cortex* (Fig. 3e). Peaks of association signatures in other populations were all shifted away from *parn*, moving towards *cortex* and its 3′ downstream region (Fig. 3e). Specifically, the association signatures of the two eastern populations, XS and SY, were located immediately downstream of *cortex*, while the western population (TR) exhibited signatures that spanned a broader region across *cortex* and its further downstream regions (Fig. 3e). Another eastern population, LY, showed top association signatures within a range similar to TR but had much fewer fixed divergence alleles (Fig. 3e). These partially overlapping yet inconsistent signatures suggest that the naturally occurring melanization in tea geometrids is constrained to this ‘hotspot’ locus but has evolved independently across different populations.

Eastern melanic populations exhibited an overall lower level of nucleotide diversity (*π*) in the ‘melanism locus’, with more evident signatures observed around *parn* and *cortex* in the LA population and the other three populations, respectively (Fig. S4). Correspondingly, the melanic morphs in these populations showed a less positive Tajima’s *D* value in this locus (Fig. S5). We also detected much lower levels of genetic differentiation (*d*_XY_) within melanic morphs of each eastern population compared to gray morphs (Fig. S6). Furthermore, Patterson’s *D*-statistics (ABBA-BABA test) showed an enrichment of shared alleles (ABBA versus BABA sites) between eastern melanic populations uniquely around the ‘melanism locus’, while this enrichment was considerably lower when analyzing gray morphs (Fig. S7). Based on the whole-genome background, these patterns suggest that the melanic morphs maintain a low level of genetic diversity specifically in the ‘melanism locus’. In addition, these patterns observed in eastern populations were largely reversed in the western population (TR) (Figs S4−S7), indicative of different evolutionary scenarios related to parallel melanism.

### Insufficient evidence linking *cortex* and *parn* to melanism of tea geometrid moths

**Figure 4.**
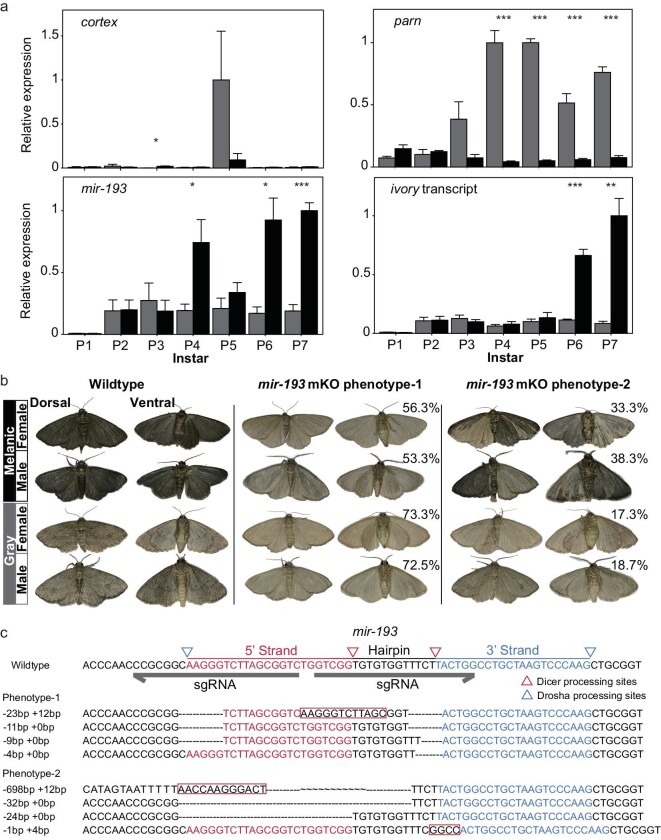
Functional investigations of causal genes within the ‘melanism locus’. (a) Expression profiles of the focal candidate genes in wing tissues of various pupal stages. Relative expression of focal genes in wing discs of a given stage is determined by qPCR. Expression levels of *parn*, *cortex* and ‘*ivory* transcript’ were normalized to the reference gene *RpS3A*, while that of *mir-193* was normalized to the small nuclear RNA (snRNA) U6. Relative expression was calculated using the 2^−ΔΔCT^ method. Mean ± SEM of three biological replicates is plotted for each morph. Melanic, black bars; gray, gray bars. P1–P7, Days 1–7 after pupation. Difference between the color morph, Student’s *t*-test: *, *P* < 0.05; **, *P* < 0.01; ***, *P* < 0.001. (b) Representative phenotypes of *mir-193* mKO mutants of tea geometrids. Phenotype of mutants with nearly complete loss of melanin across all wings was termed phenotype-1, while those showing softened depigmentation were termed phenotype-2. The percentage value in the top-right represents the proportion of the corresponding phenotype among all mKO mutants. (c) Genotyping information of representative *mir-193* mKO mutants. Two single guide RNA (sgRNA) target sites indicated by arrowheads were designed for targeting both the Drosha and Dicer processing sites. The numbers on the left of each sequence indicate inserted (+) and/or deleted (−) bases in comparison to wild type. Insertions are highlighted in red.

The results of linkage mapping and GWAS suggest that the broad genomic region encompassing *cortex* and *parn* has a universal effect on melanism. We examined the temporal expression profiles of these two protein-coding genes in wing tissues throughout pupal development. The expression of *cortex* was low overall in both morphs across most pupal stages, with the exception of Day 5, when a peak with high variance was observed in the gray morph (Fig. 4a). On the other hand, the expression of *parn* showed differential patterns between the two morphs during the mid to late pupal stages, due to a dramatic increase unique to the gray morph since Day 3 (Fig. 4a). If *parn* is indeed the causal effector, its role would be associated with depigmentation.

We further performed CRISPR/Cas9-based loss-of-function experiments to test the coloration phenotypes of these two coding genes. Mutagenesis of *cortex* did not yield any viable offspring with effective mutations, which may be attributed to the known roles of *cortex* in meiosis and embryonic development [17,18]. Alternative attempts in the model species of Lepidoptera, *Bombyx mori*, were able to produce mosaic knockout (mKO) mutants, but they were not observed with any visible morphological changes. Further self-mating between the mKO mutants failed to produce viable offspring, likely due to the fertility effects observed in female mutants (Fig. S8a). We also attempted to overexpress *cortex* in *B. mori* using a UAS/Gal4 transgene system. This resulted in conspicuous melanization in the fourth-instar larvae (Fig. S8b), but this unexpected larval melanism vanished in the subsequent developmental stage, along with a developmental retardation (Fig. S8b).

Gene mutagenesis of *parn* in tea geometrids produced a few eclosed adults with mutations that effectively disrupt protein coding (Fig. S9b). Of these mKO mutants, we observed different wing phenotypes, including small patches of reduced melanization in the ventral right forewings and a pinch effect in the hindwings (Fig. S9a). Notably, these phenotypes are distinct from those reported in butterflies [19]. Importantly, the abatement of melanization observed upon knockout contradicts the expression pattern of *parn* that supports a role in depigmentation (Fig. 4a; Fig. S9).

### *mir* *-193* is the likely melanism regulator in tea geometrids

While this work was in progress, studies on butterflies reported that a long non-coding RNA (ncRNA), *ivory*, and its proposed functional product, *mir-193*, rather than *cortex* itself, serve as the functional effectors of the ‘*cortex* locus’ [19–21]. We began our investigations in tea geometrids by focusing on *mir-193*, as its function in regulating melanic coloration has been verified in both butterflies and *Drosophila melanogaster* [19]. The homolog of *mir-193* was identified in the ‘*cortex* locus’, 3.6 kb downstream of *parn*. The expression of *mir-193* was relatively stable in the gray morph during pupal stages, while its expression in the melanic morph exhibited evident peaks on Days 4, 6 and 7, which were significantly higher than those observed in the gray morph (Fig. 4a). To examine its phenotype, we functionally blocked the biogenesis of mature *mir-193* by targeting both the Drosha and Dicer processing regions. Strikingly, we observed clear phenotypic changes in the mKO mutants of both morphs, with over half of them showing a nearly complete loss of melanin across all wing parts (Fig. 4b and c). In addition, a small proportion of the mutants exhibited incomplete depigmented patterns (Fig. 4b). Genotyping results revealed that at least one strand of the microRNA (miRNA) and the associated Drosha and Dicer processing sites remained intact in these mutants (Fig. 4c).

*mir* *-193* was considered the functional unit of the long ncRNA *ivory* [19]. *Ivory* shows no sequence homology even across closely related species [19,20]. Indeed, we only identified a genomic segment homologous to its core promoter region (termed as *ivory_P* hereafter) in the tea geometrid genome, which is located upstream of *cortex* (Fig. S10). To annotate the transcripts of *ivory* and other potential ncRNAs, we performed high-coverage transcriptome sequencing of larval and pupal wings from both melanic and gray morphs (see Materials and Methods). *De novo* assembly of all the data predicted 30 transcripts within the ‘melanism locus’ (Fig. S10; Table S7), including *parn*, which had the highest abundance, and *cortex*, which had with trace abundance (Fig. S10; Table S7). It is noteworthy that one of the assembled transcripts is located immediately adjacent to *ivory_P*, although both its length and the genome it spans are considerably shorter than those of butterflies (Table S7).

Interestingly, this candidate *ivory* transcript was detectable by quantitative PCR (qPCR) and showed an expression pattern similar to that of *mir-193*, particularly the peaks observed during the late pupal stages that are unique to melanic morphs (Fig. 4a). For the gene mutagenesis experiment, we specifically targeted its conserved promoter, as this is the only convincingly annotated region and has direct functional evidence in butterflies [19–21]. As a result, we observed a melanin-loss phenotype in the *ivory_P* mKO mutants, although the effect was less pronounced compared to that of the *mir-193* mutants. For instance, the depigmentation phenotype was limited to certain areas and occurred at relatively low frequencies in the mutants of the melanic morph (Fig. S11a). In the gray morph, the depigmentation effect upon knockout of *ivory_P* was even less noticeable, with only the distal margins showing visible differences between the mutants and wild-type (Fig. S11a). Thus, our results reveal that, as the potential functional product of *ivory*, *mir-193* is the major effector of the melanism locus in tea geometrids.

### Possible selective disadvantage on melanization in tea geometrids

**Figure 5.**
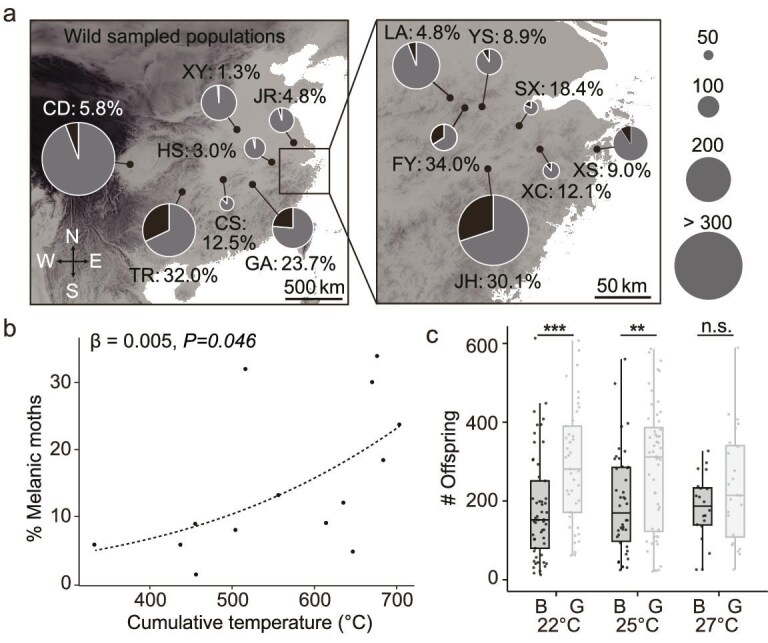
Ecological consequences of melanism in tea geometrids. (a) Proportions of melanic morphs of tea geometrids at different sampling sites. Due to the dense sampling sites in Zhejiang, China, this region is shown in an enlarged view on the right. The size of pie charts indicates the total number of sampling moths with definite phenotypes. The proportion of the melanic morph (shown in black) for each population is indicated along with the pie chart. (b) Correlation between the temperature condition (33-day period) and the ratio of melanic morphs. Each dot indicates a population with field data. See detailed information in Table S8. (c) Fecundity comparison between the melanic (B) and gray (G) morphs under different temperature conditions. Each dot indicates the total survival numbers of offspring of a mating pair. The box plot indicates the interquartile range. Wilcoxon test: *, *P* < 0.05; **, *P* < 0.01; ***, *P* < 0.001. Review drawing number: GS京 (2025) 2216号.

It is interesting to understand the potential ecological factors driving the evolution of melanism in tea geometrids, particularly due to the apparent absence of strong natural selection such as the changing environment color that disrupts the benefits of camouflage or mimicry (Fig. 1). To do this, we investigated the frequencies of melanic morphs in the field by trapping tea geometrids across 14 different tea-cultivated locations (Table S8). We found that, despite being the dominant phenotype, melanic morphs constituted a minor proportion of the population, ranging from 1.3% to 34.0% in each location investigated (Fig. 5a). Interestingly, melanic morphs were either maintained at a lower ratio or were nearly absent in northern populations (Fig. 5a), leading us to test the potential association between melanization and temperature adaptation in this species. We used the effective cumulative temperature over a 33-day period—approximately the duration of a complete generation of tea geometrids—as a measure of temperature at each sampling site, and showed a positive correlation between this temperature factor and the local proportion of melanic morphs (β = 0.005, *P* = 0.046; Fig. 5b). This significant correlation could suggest that higher temperature might be relatively advantageous for melanic tea geometrids in certain contexts. To further test this hypothesis, we compared the fecundity of melanic and gray morphs under different temperature conditions. Our results indicated a significant relationship between fecundity and morph type (*LR*χ^2^ = 7.03, *P* = 0.008), while temperature (*LR*χ^2^ = 1.56, *P* = 0.46) and the interaction between morph and temperature (*LR*χ^2^ = 0.80, *P* = 0.67) did not show significant correlations (Table S11). Specifically, the gray morph produced significantly more offspring than the melanic morph at 22°C (pairwise *post hoc* multiple comparisons; *z* = 3.374, *P *= 0.0007) and 25°C (*z* = 2.675, *P *= 0.0075) (Fig. 5c; Table S11). When the temperature increased to 27°C, while gray moths still produced a higher number of offspring on average, the difference was no longer statistically significant (*z* = 1.246, *P *= 0.21; Fig. 5c; Table S11). These observations jointly suggest a slight selective disadvantage for melanism in terms of reproduction.

In addition to latitude, temperature also varies with the seasons. We established eight sampling sites to continuously collect population data across consecutive seasons (i.e. spring, summer, autumn and winter) from the same locations over months (Table S8). However, statistical analysis showed no significant differences in the proportion of melanic morphs among the populations in each season (Fig. S12b), with all populations being dominated by a low frequency of melanic forms (Kruskal–Wallis tests, χ² = 1.28, *P *= 0.73) (Fig. S12a). A possible explanation would be that the differences in temperature adaptation between melanic and gray morphs have not led to seasonal effects on regulating the clines of tea geometrids. Previous studies on arthropods have suggested an integrated effect of temperature and precipitation (humidity) affecting the frequency and dynamics of melanic polymorphism [22,23]. Our investigations showed no significant correlation between precipitation and the proportion of melanic morphs in tea geometrid populations (Fig. S13).

## DISCUSSION

Elucidating the genetic and ecological mechanisms underlying phenotypic innovation and diversification is a central theme in evolutionary biology. Over the past decade, a handful of genetic loci associated with color variation have been identified in Lepidoptera, particularly in certain lineages of butterflies [24]. Remarkably, even in a single genus, at least four master switch loci (*wntA*, *optix*, *aristaless1* and *cortex*) have been linked to variations in wing color in *Heliconius* butterflies across the Neotropics [11,25–27]. In contrast, despite constituting the majority of described Lepidoptera, to our knowledge, the *cortex* locus is the only one that has been identified for color variations in moths. This locus simultaneously controls the natural body color switch between melanic and gray in various Geometridae moths, spanning approximately 30 million years of evolution across both western and eastern Eurasia [5,10], as well as the wing pattern variation in Erebidae [28]. It seems that color variation in moths is largely constrained to this hotspot region, which has been repeatedly used by unrelated lineages.

However, the process of identifying the causal effector within this melanism locus is complex and torturous. Initially, the gene *cortex* was proposed as the causal effector within this locus and has been the focus of many subsequent studies [5,11,13,14,29], although some studies occasionally mentioned its flanking regions, including the 5′ extensions toward the gene *parn* or several closely arrayed trivial genes at the 3′ region [11,24,29]. The molecular function of *cortex* in *Drosophila* is to regulate meiosis and the cell cycle in ovaries [17,18]. Most presented functional evidence supporting *cortex* as a color switcher in lepidopterans is marginal, including the differential gene expression at specific developmental stages and the occasional presence of small depigmented scales in the wings of mKO mutants [14,30,31]. In our functional examinations involving tea geometrids and silkworms, we found no evidence linking *cortex* to adult pigmentation. Instead, we observed partially lethal effects from mosaic mutagenesis, as well as temporary pigmented phenotypes in larvae upon overexpression (Fig. S8).

Recent studies on butterflies have rewritten our understanding of the *cortex* locus, concluding that the long ncRNA *ivory*, and its derived functional product *mir-193*, serve as the major effector. These ncRNAs showed expression that prefigures adult melanic patterns and high penetrance of depigmentation phenotypes in KO mutants across different butterfly species [19–21]. This convincing evidence has also prompted us to test their roles in moth melanism. In this study, we generated mKO mutants for both color morphs, with a substantial proportion displaying a marked loss of melanin on all scales (Fig. 4b). We also found that the temporal expression pattern of *mir-193* in melanic moths closely resembled that of butterflies exhibiting melanic scales on most areas of their wings (Fig. 4a). These results support the causal role of *mir-193* in moth melanism.

The long ncRNA *ivory* was considered to function as a scaffold (i.e. a primary miRNA) for the expression and processing of *mir-193* [32]. However, while *mir-193* is deeply conserved across insects, *ivory* is likely evolving rapidly, because there is no clear sequence homology for this long ncRNA across Lepidoptera, except for the approximately 100-bp core promoter region [20]. Disruption of the homologous region of the *ivory* promoter indeed resulted in reduced pigmentation in adult tea geometrids, but the strength of this reduction is much weaker compared to the effects observed when *mir-193* was knocked out in this species (Fig. 4; Fig. S11) or when *ivory* was knocked out in butterflies [19–21]. We also note that the identity of the *ivory* transcript characterized in this study is somewhat unconvincing, as it largely differs from those identified in butterflies in terms of sequence, exon–intron structures, and the genomic regions it spans. The temporal expression of this transcript in tea geometrids also diverges from that documented in the *Bicyclus anynana* butterfly. Specifically, the expression in tea geometrids peaks at the end of the pupal stage (Fig. 4a), synchronizing with *mir-193*, rather than occurring earlier than *mir-193*, as seen in *B. anynana* [19], which could support the notion that *mir-193* is derived from *ivory*. Therefore, it remains unclear for us whether this transcript is the actual *ivory*, whether it plays a similar role to the *ivory* in butterflies, or whether there are other unidentified regulatory elements in moths that control the formation of *mir-193*; the presence of multiple regulators could help explain the universal genotype–phenotype association effects throughout the broad *cortex*-*parn* region.

Another protein-coding gene within this locus, *parn*, has also received our attention for its strong association with trait segregation in the cross-mapping progeny (Fig. 2). *parn* is located distantly from *cortex* but adjacent to *mir-193* (Fig. 2e). This gene has only been mentioned in a few previous studies for mapping color polymorphisms in certain butterfly species, but it has not been subjected to in-depth investigations [11,24,29]. The described functions of *parn* include degrading the poly(A) tails of eukaryotic messenger RNAs (mRNAs) and helping the maturation of small RNAs in the silkworm [33–35]. In this study, we observed marked differential expression of *parn* between the morphs, with upregulation in the gray morph since the mid pupal stage (Fig. 4a). However, this pattern is contrary to those of *mir-193* and *ivory*, making it difficult to tie to the expression and processing of this functional unit.

Interestingly, the role of *mir-193* in melanin production challenges the traditional notion of using ‘melanization’ to depict color variations in moths. This pigmentation effector has ancient origins and is deeply conserved across insects, suggesting that producing melanin pigment is more likely the ancestral state, rather than a trait that was independently derived in various insect lineages. Thus, a likely scenario would be that mutations occurred independently in this hotspot region, some of which functionally disrupted this pri-lnc-miRNA complex or its processing pathway, repeatedly leading to depigmentation in light-colored morphs of various species (e.g. the gray morph of tea geometrids). Indeed, we found that *mir-193* was not activated in the gray morph as it was in the melanic morph (Fig. 4a), resulting in differential expression between the color morphs during the mid to late pupal stages.

Nevertheless, the gray–melanic polymorphism of tea geometrids provides an opportunity to explore how color variations have been shaped by a combination of multiple ecological factors, each having slightly selective advantages or disadvantages. For example, while melanization is a general sign of immune responses that are effective against pathogens, this process incurs substantial energetic cost, leading to abnormal development or reduced fecundity in insects [36–38], and plays less important roles in the adult stage. In addition, despite the dominant phenotypic effect of the melanic allele over the gray allele, field investigations reveal that melanic morphs of tea geometrids account for a minor proportion in all observed wild populations (Fig. 5a), agreeing with the discovery of relatively limited genetic diversity in melanic morphs (Figs S4−S6). These observations from molecular and ecological perspectives could be explained by the synthesis of fitness, genetic variation and proposed evolutionary scenarios. First, the higher frequency of gray morphs indicates a selective advantage for this recessive allele. We have performed reproductive assays that showed reduced fecundity in the melanic morph of tea geometrids (Fig. 5c). This reproductive disadvantage in fecundity is somewhat mitigated under high-temperature conditions, which might explain the increased ratios of melanic morphs observed in warmer geographic locations (Fig. 5a). Interestingly, this temperature-dependent fitness of melanism in tea geometrids is contrary to a thermal melanism hypothesis for moths, while partially agreeing with the ecogeographical Gloger’s rule [39–41]. Second, the reuse of a single locus in independent evolutionary events could be attributed to specific DNA characteristics that exhibit a high rate of variation [13]. Indeed, this ‘melanism locus’ has high levels of variations in tea geometrids (Fig. S14). Simultaneously, we did not detect clear evidence of balancing selection within the ‘melanism locus’ in tea geometrid populations (Fig. S15). Thus, the high frequency of variations within this hotspot might enable the repeated disruptions of the melanic-making function, leading to the emergence of genetically diverse gray morphs. Taken together, our study empirically demonstrates that a variation hotspot not only contributes to adaptive evolution but also enables non-adaptive phenotypic diversification.

## MATERIALS AND METHODS

### Cross-mapping of the melanism locus

The gray and melanic morphs of tea geometrids (*E. grisescens*, Warren 1894) were collected in Yangzhou, China (119°12′E, 32°18′N) and Shaoxing, China (120°34′E, 29°16′N), respectively. All lines of tea geometrids were reared in the laboratory conditions of 25°C ± 1°C, 75%–80% humidity and 13:11 light/dark photoperiod. Melanic and gray moths were hybridized to produce a backcross mapping population, which consisted of 904 individuals (457 melanic and 447 gray; Table S2). DNA was prepared individually from the whole body of the adult stage. All DNA samples were randomly grouped and equally pooled into 20 mixed samples for standard Illumina sequencing (Table S4). A bulked segregation analysis (BSA) approach was applied to identify causal loci based on allele frequencies of the two morphs.

### Population genetic analysis of wild populations

Wild populations exhibiting both color morphs were sampled from five main producing areas of tea in China (Table S6). Eight individuals of each morph were subjected to whole-genome DNA sequencing for each population (Table S6). Sequencing data of each sample were mapped to the reference genome of *E. grisescens* [42]; variants were called using a standard Genome Analysis Toolkit (GATK) pipeline [43]. High-confidence, bi-allelic variants were filtered for subsequent population genetics analyses.

### Functional investigations of causal genes

Transcriptomes of larval and pupal wing tissues from both color morphs were sequenced to annotate genes in the candidate locus and to compare the expression profiles between the morphs. Reverse transcriptase quantitative polymerase chain reaction (RT-qPCR) was conducted to analyze the expression profiles of candidate genes, including *cortex*, *parn*, *ivory* and *mir-193*. To test the function *in vivo*, CRISPR/Cas9-based gene mutagenesis was applied for each of these candidates in tea geometrids, following the procedures described [42].

### Occurrence observation in the field

Field investigations were conducted to document the frequency of each color morph across 14 tea-producing areas in China since 2019 (Table S8). Cumulative temperature and precipitation of a generation (∼33 days) were calculated for each population, in which over-wintering populations (i.e. the first generation of every year) were excluded from the analysis. Relationship between cumulative temperature and the proportion of melanic morphs was analyzed through generalized linear model (GLM) in R with quasibinomial distribution.

See Supplementary data for more detailed information.

## Supplementary Material

nwaf441_Supplemental_Files

## Data Availability

The datasets generated in this study have been deposited in the Sequence Read Archive (SRA) with BioProject accession PRJNA1169944 (for cross-mapping), PRJNA1170005 (for population genetics of wild populations) and PRJNA1170008 (for transcriptome sequencing).
