# Supplementary material for: The genetics and evolution of moth melanism in the absence of strong natural selection
Source: Natl Sci Rev. 2025 Oct 23;13(1):nwaf441. doi: 10.1093/nsr/nwaf441 (PMC12796802; doi:10.1093/nsr/nwaf441)
Supplement: nwaf441_Supplemental_Files [file nwaf441_supplemental_files.zip › Supplementary_figures.pdf]

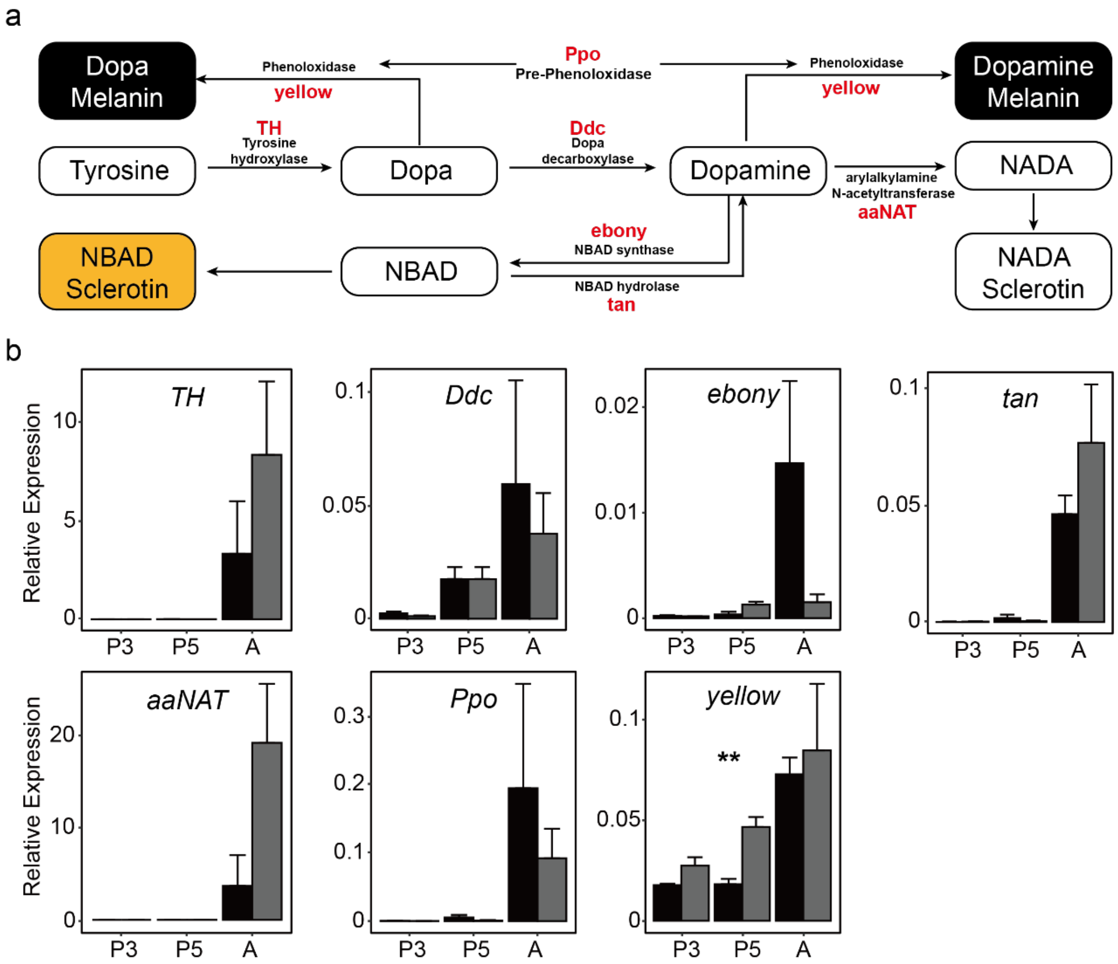

Figure S1 Expression patterns of melanin synthesis-related genes between the grey and melanic morphs. (**a**) Schematic diagram of the melanin synthesis pathway in insects. (**b**) Relative expression of each focal gene in pupal (P3, day 3 pupae; P5, day 5 pupae) and adult (A) wings. Expression is quantified by qPCR and normalized to ribosomal protein *RpS3A* as a reference. Data was plotted with mean ± S.E.M. for each comparison, in which melanic and grey morphs are shown in black and grey, respectively. Student's *t* test: ** $P < 0.01$.

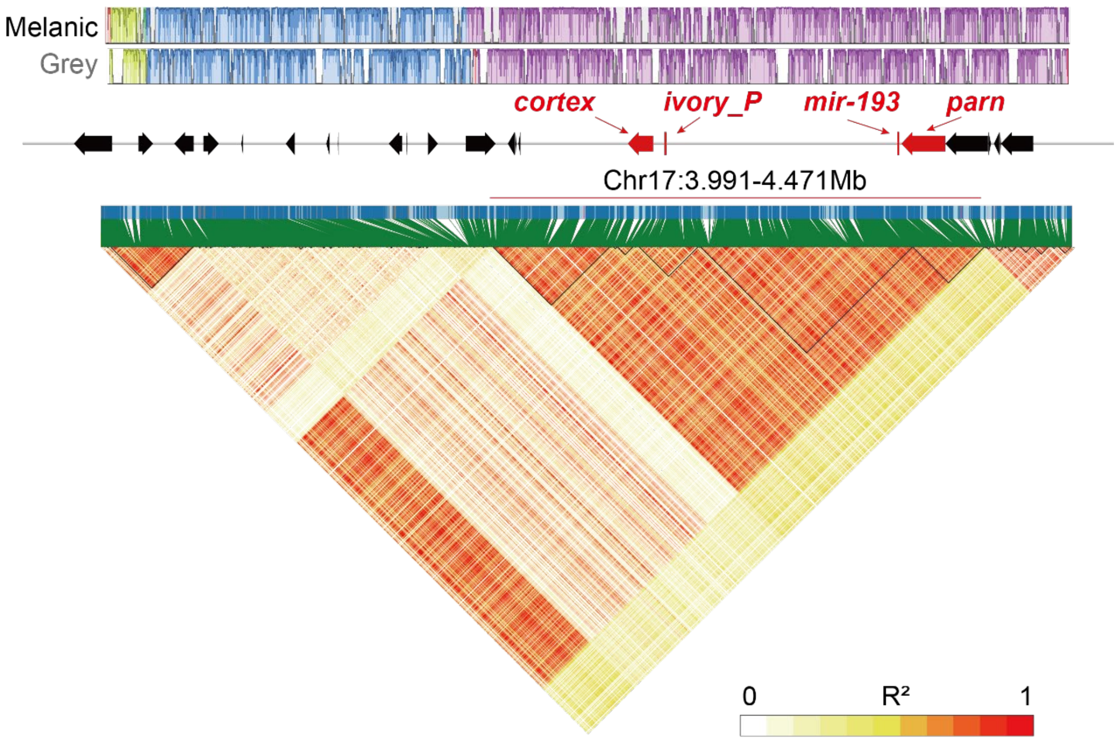

Figure S2 Local alignment and linkage disequilibrium around the candidate melanism region. Top, syntenic blocks determined by the alignment between the local assemblies of melanic and grey morphs. Blocks in the same color indicate homologous alignment and the height of the blocks indicates the average sequence similarity of the corresponding genomic region. Non-aligned or poorly aligned regions (in white) indicate potential structural variations (SVs). Bottom, pairwise linkage disequilibrium is estimated as the square of the correlation coefficient between SNPs within the mapping progeny (R^2). The interval underlined by the red line indicates the 'melanism locus'.

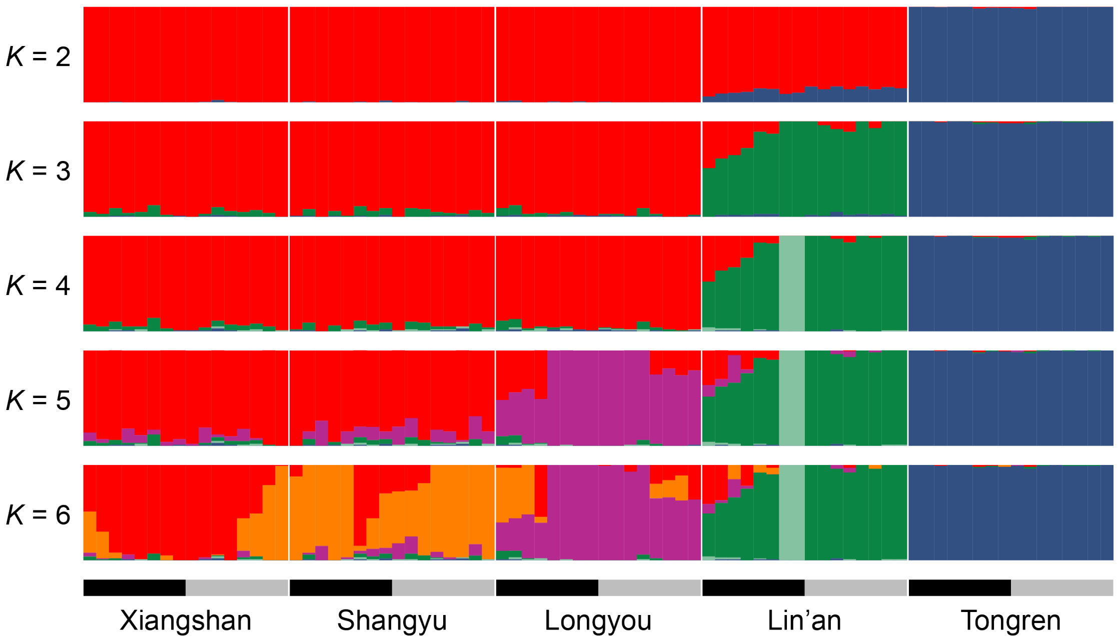

Figure S3 Population structures of wild tea geometrid individuals based on ancestry estimation. The color in each column represents the ancestry proportion, with presumed group sizes from $K = 2$ to 6. Individuals with grey or melanic morph are grouped by the bottom bar.

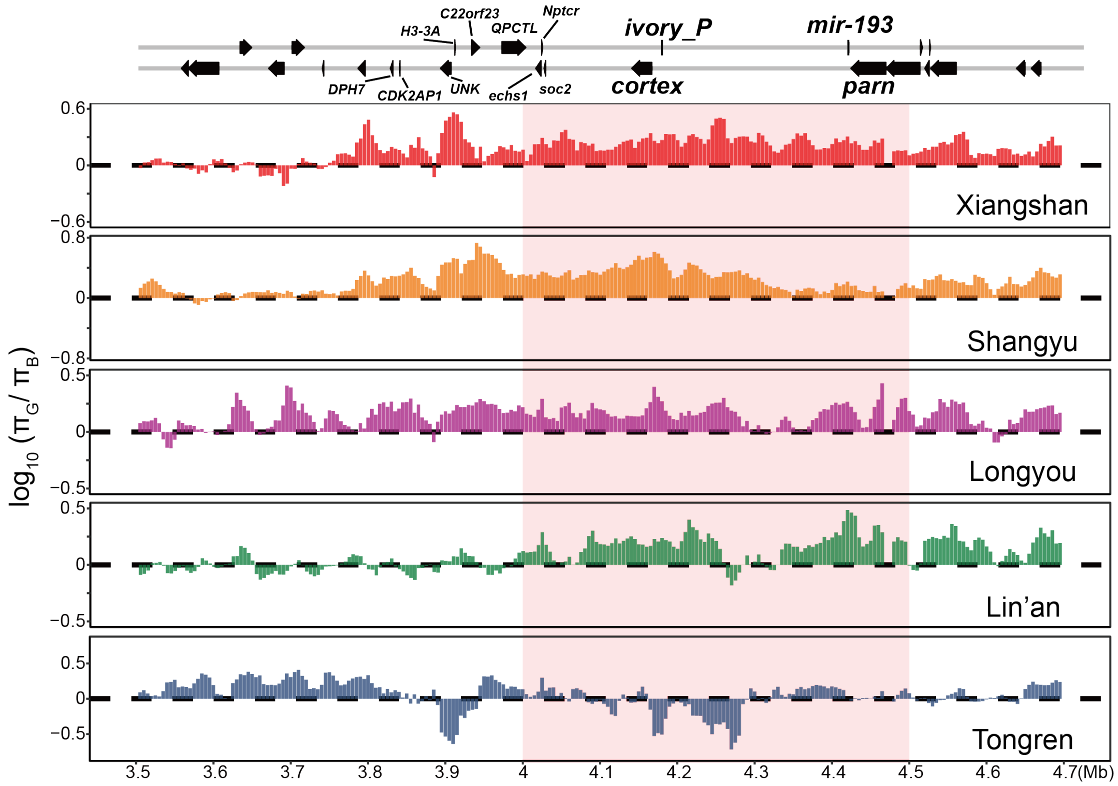

Figure S4 Comparison of nucleotide diversity between the color morphs for each population. The nucleotide diversity (π) of a 20-Kb window, with 5-K step length, is compared between the grey (G) and melanic (B) morphs, whose ratios in log-transformed are indicated as histograms along with the 'melanism locus' labeled by pale red shadow. The positive values indicate higher level of π in the grey morph of the focal population.

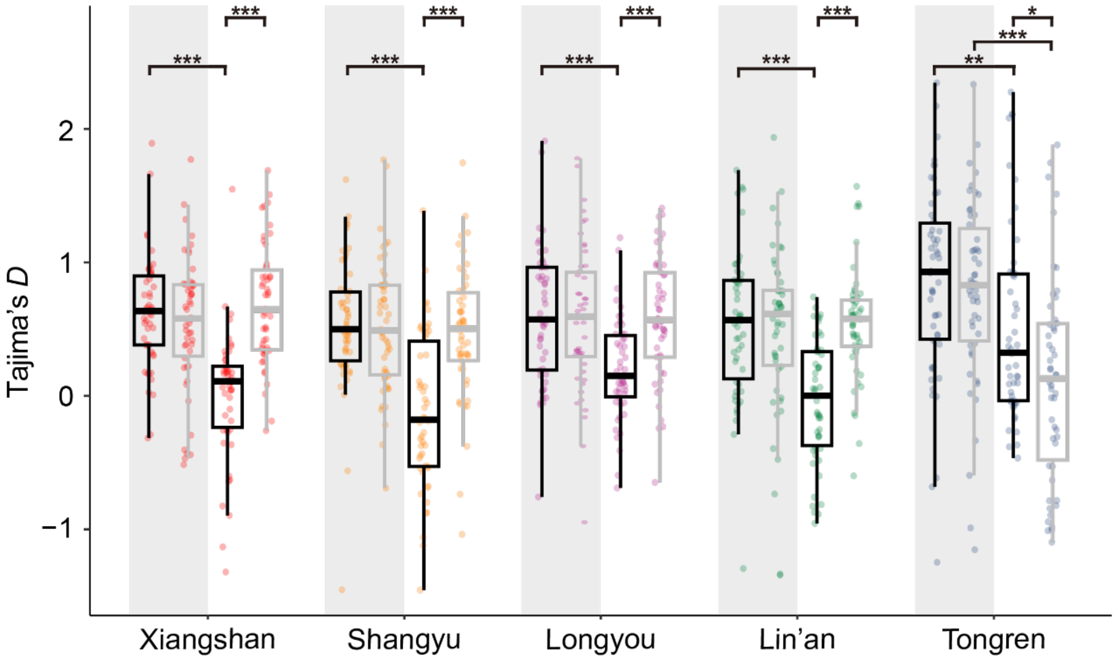

Figure S5 Comparison of Tajima's *D* between the two morphs of each population in the 'melanism locus' and the whole genome background. Tajima's *D* of a 10-Kb genomic window are compared between the grey (outlined by grey boxes) and melanic (outlined by black boxes) morphs in 'melanism locus' versus a randomly sampled genome-wide background (shaded) of equivalent size, where jittered scatter plot and boxes represent the distribution and quartile ranges for each group of tea geometrids. Student's *t* test: *, $P < 0.05$; **, $P < 0.01$; ***, $P < 0.001$.

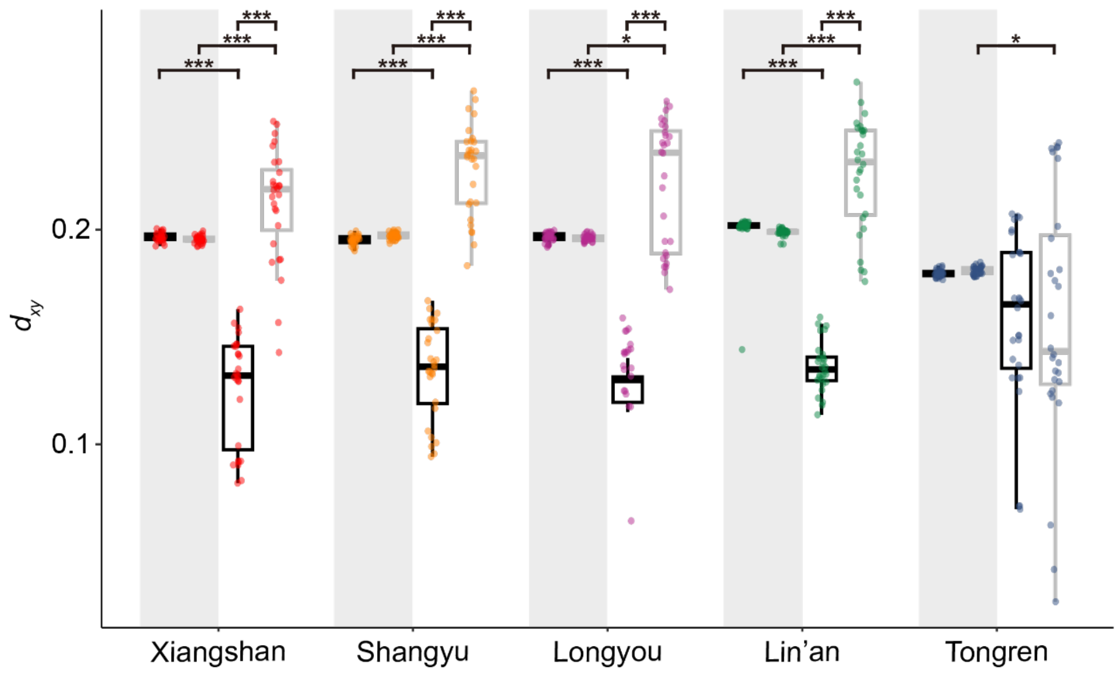

Figure S6 Comparison of d_{xy} between the two morphs of each population in the 'melanism locus' and the whole genome background. d_{xy} in 'melanism locus' versus genome-wide background (shaded) was calculated between pairs of individuals within grey (outlined by grey boxes) and melanic (outlined by black boxes) morphs, where jittered scatter plot and boxes represent the distribution and quartile ranges for each group of tea geometrids. Student's *t* test: *, $P < 0.05$; ***, $P < 0.001$.

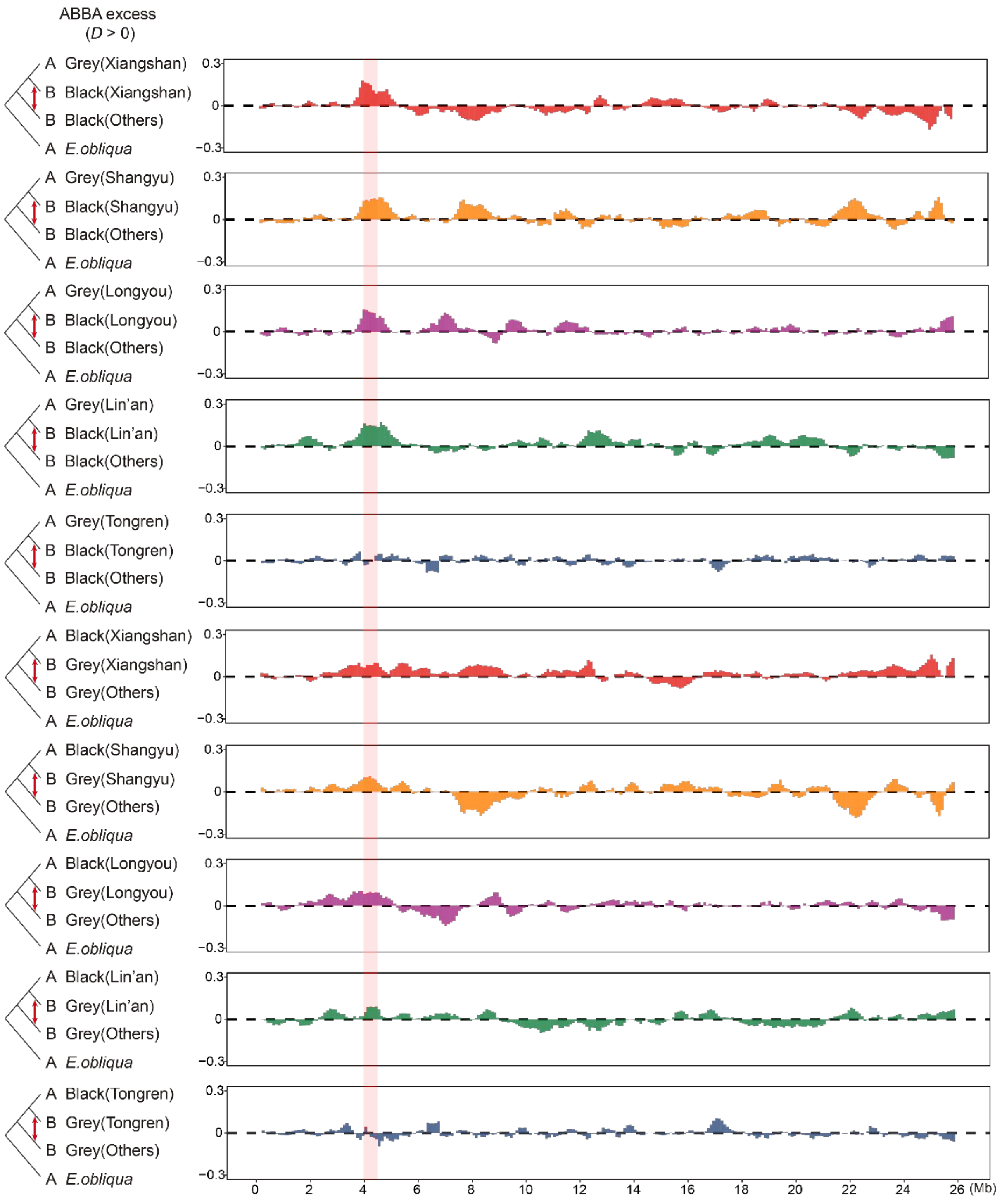

Figure S7 Analyses of shared alleles between the melanic and grey morphs in various populations. Patterson's *D* (ABBA-BABA) statistics of a 500-Kb window, with 100-Kb step length, is indicated as histograms along with Chromosome 17, with the 'melanism locus' highlighted in the red shadow. The phylogeny for ABBA-BABA test is indicated on the left. A positive *D* indicates the strength of shared alleles between two centered population. *E. obliqua* is a closely relative species of *E. grisescens*, which is used as outgroup.

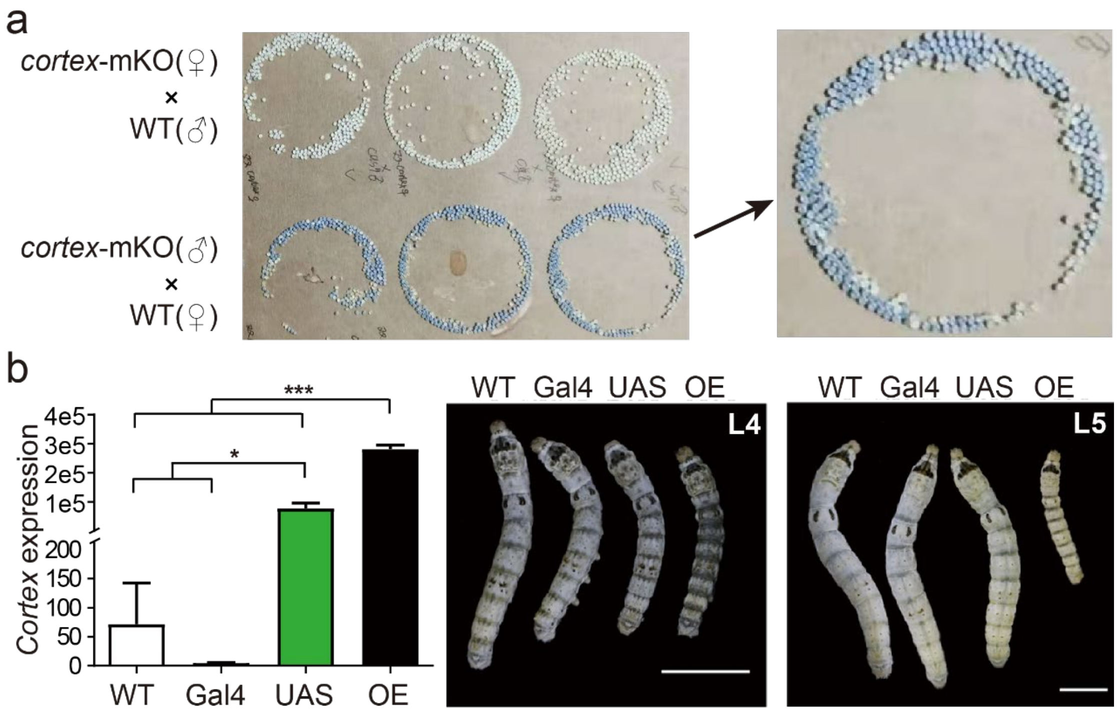

Figure S8 Functional investigations of *cortex* in silkworms. (**a**) Embryonic development of offspring of *cortex* mosaic knockout mutants (mKO). The photos show the condition of laid eggs by indicated parents on day 10 after oviposition. The darkened eggs indicate those under normal development. (**b**) Overexpression of *cortex* in *B. mori* using the binary Gal4/UAS transgenic silkworm system. From left to right: the expression of cortex examined in the epidermis of various lines (mean ± S.E.M.), the morphology from dorsal side of the fourth-instar larvae (L4), and the morphology from dorsal side of the fifth-instar larvae (L5). WT, wild type; Gal4, the Gal4 line detected with DsRed2; UAS, the UAS line detected with EGFP; OE, the crossed line between Gal4 and UAS indicating the positive strain with overexpression of cortex. Student's *t* test: *, $P < 0.05$; ***, $P < 0.001$. White line, 1 cm.

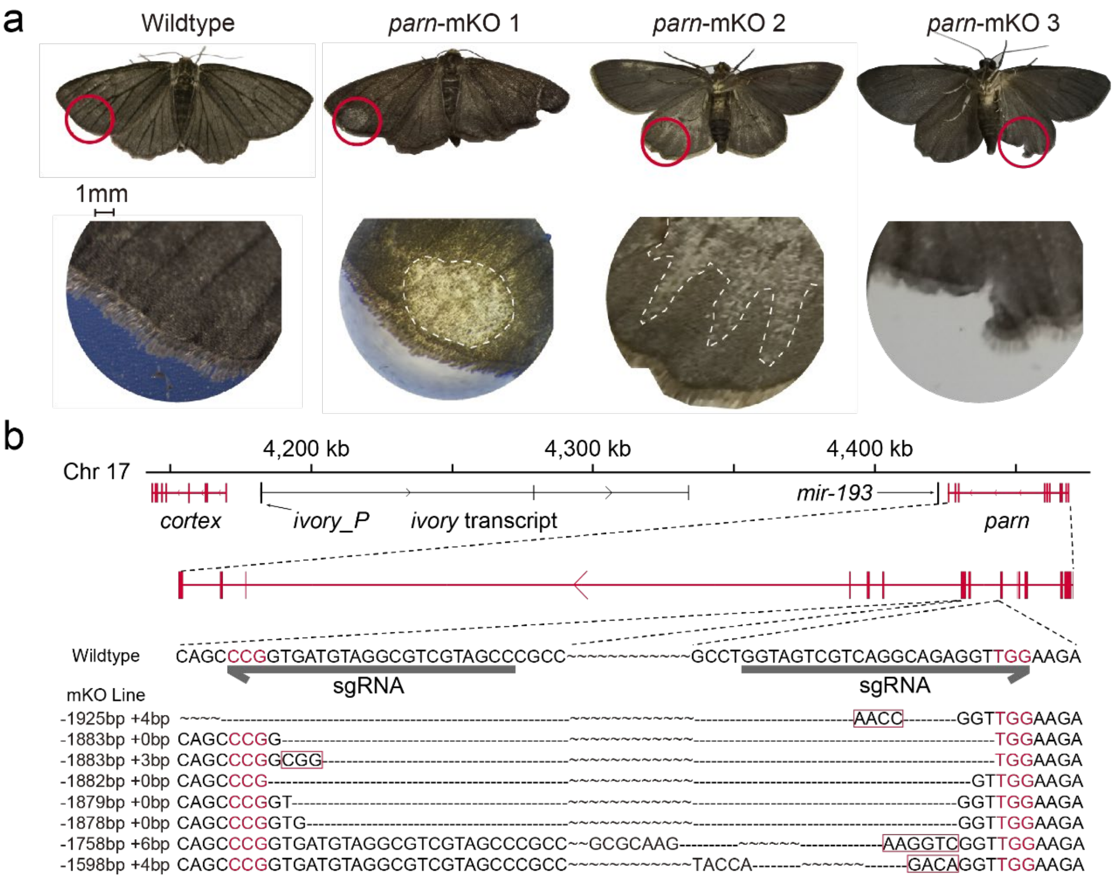

Figure S9 Gene mutagenesis of *parn* in tea geometrids. (**a**) Representative phenotypes of *parn* mosaic knockout mutants (mKO). The enlarged view of the framed region is shown below. White dashed lines indicate the boundaries of the abnormal wing color. Note that the phenotype shown as mKO1 is not a classical coloration phenotype as reported for mKO in butterflies (e.g., [19]). (**b**) Genotyping information of representative *parn* mKO mutants. Two sgRNA target sites were indicated by arrowheads, with PAM sequence highlighted in red. The numbers on the left of each sequence indicate inserted (+) and/or deleted (−) bases in comparison to wild type. Insertions are highlighted inside red boxes.

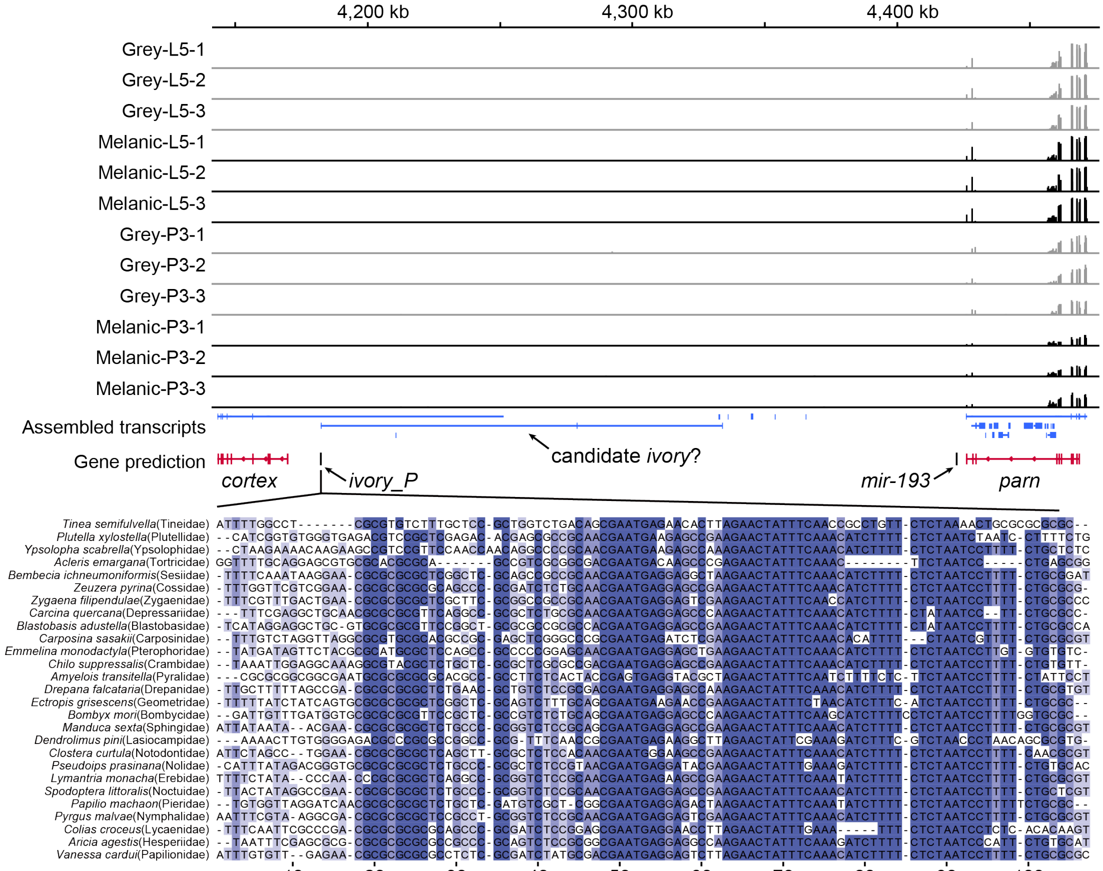

Figure S10 Annotation of non-coding RNAs within the 'melanism locus' based on wing transcriptome sequencing. The histograms in the top panel display the mapping coverage of 12 sequenced transcriptomes of larval and pupal wing tissues of both melanic and grey morphs. Melanic-L5-1-3, the three replicates of wing discs of 5th-instar larvae of the melanic morph; Melanic-P3-1-3, the three replicates of wing tissues of day 3 pupae of the melanic morph; Grey-L5-1-3 and Grey-P3-1-3 indicate the corresponding samples of the grey morph. The architectures of *de novo* assembled transcripts are shown along with chromosomal positions as below. The bottom panel display multiple sequence alignment of the putative *ivory* promoters across representative lepidopteran species. Sequences of species rather than *Heliconius melpomene*, *Heliconius erato*, and *Bicyclus anynana* are identified from the reference genome of the given species. Shadows from white to dark blue indicate the increases in conservation among species.

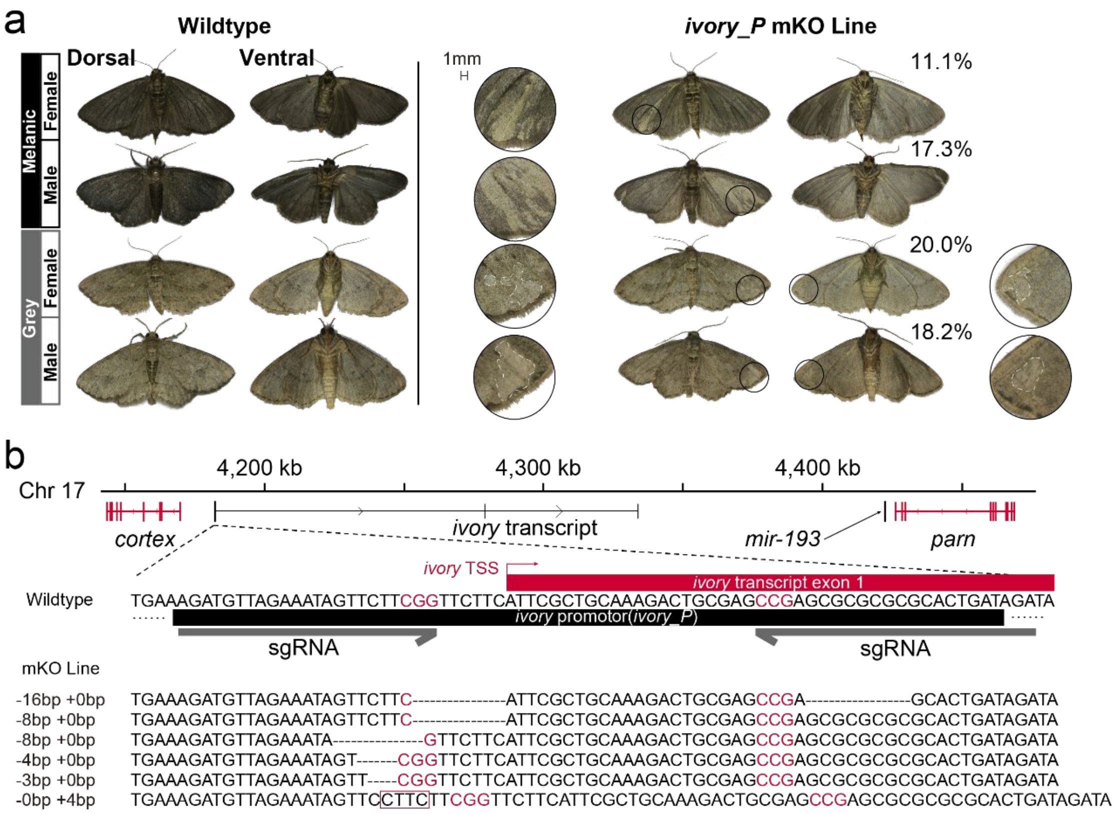

Figure S11 Gene mutagenesis of the candidate *ivory* promoter in tea geometrids. (**a**) Representative phenotypes of mosaic mutants (mKO) by disrupting the candidate promoter region of *ivory* (*ivory_P*). The enlarged view of the framed region is shown at each side. White dashed lines indicate the boundaries of the abnormal wing color. Phenotypic ratios are shown on the top-right. (**b**) Genotyping information of representative *ivory_P* mKO mutants. Two sgRNA target sites were indicated by arrowheads, with PAM sequence highlighted in red. The numbers on the left of each sequence indicate inserted (+) and/or deleted (−) bases in comparison to wild type. Insertions are highlighted inside red boxes.

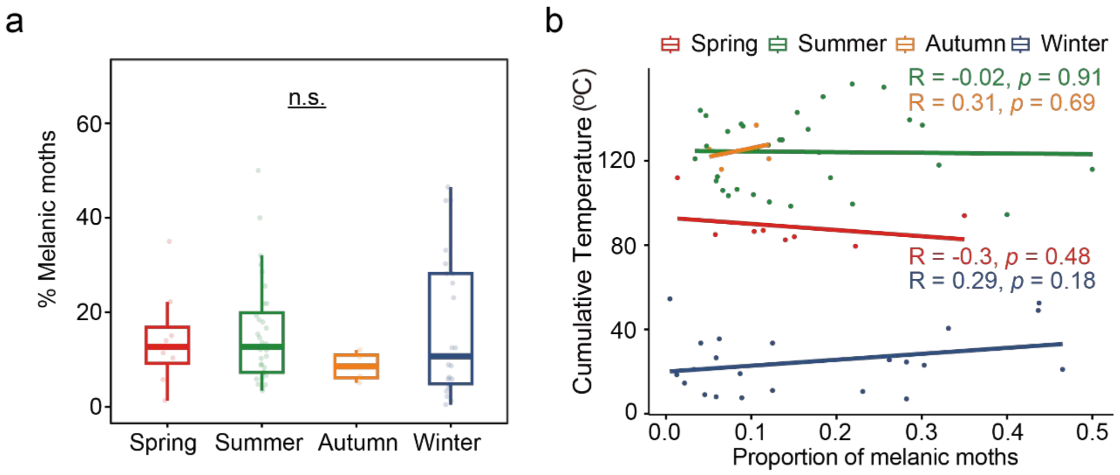

Figure S12 Seasonal effects on the frequency of melanic morphs in different populations. (**a**) Observed ratios of melanic morphs across consecutive seasons (spring, summer, autumn and winter) of different sampling sites. The box plot indicates the interquartile range. Wilcoxon test shows no significant difference between the morphs. (**b**) Correlation analysis for 7-day cumulative temperature and black ratio in populations across consecutive seasons. See sampling information in Table S8.

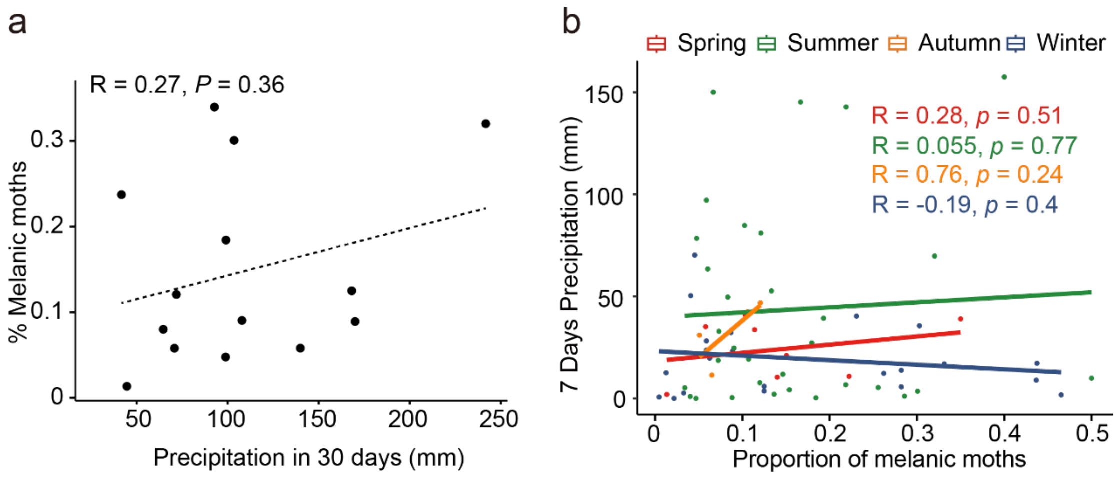

Figure S13 Precipitation effects on the frequency of melanic morphs in different populations. (**a**) Correlation between the precipitation and the ratio of melanic morphs. Each dot indicates a population with field data of cumulative precipitation within a complete lifecycle of the tea geometrid (~33 days) and the observed ratio of melanic morphs. (**b**) Correlation analysis for 7-day cumulative precipitation and black ratio across consecutive seasons (spring, summer, autumn and winter). See sampling information in Table S8.

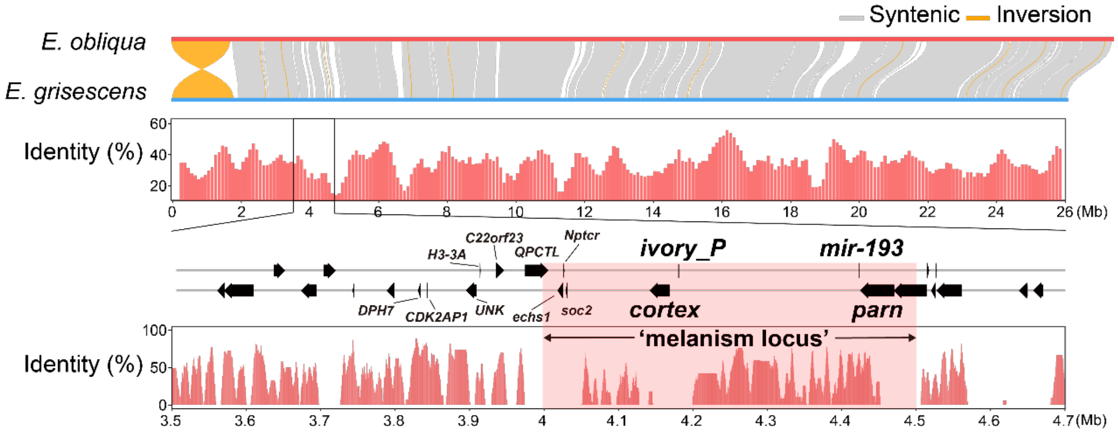

Figure S14 Genomic divergence between the tea geometrid and its closely related species (*E. obliqua*) on chromosome 17. Top, visualization of chromosome-level homologous regions between the two sibling species. Syntenic and inverted region was labeled by grey and yellow respectively, which white gap means un-aligned regions. Corresponding sliding-window mapping identity was illustrated as below, along with the enlarged view of the 'melanism locus'.

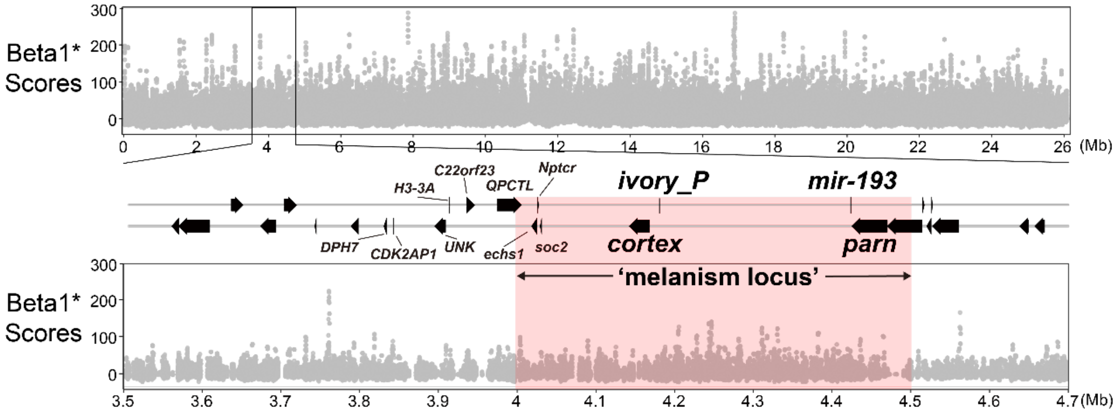

Figure S15 Detection of balancing selection signal in tea geometrid populations. Data on chromosome 17 is shown. Beta1* scores of each variation were plotted against coordinates. Higher values indicate likely evidence of balancing selection. The enlarged view of the 'melanism locus' was illustrated as below.
