# Supplementary material for: The genetics and evolution of moth melanism in the absence of strong natural selection
Source: Natl Sci Rev. 2025 Oct 23;13(1):nwaf441. doi: 10.1093/nsr/nwaf441 (PMC12796802; doi:10.1093/nsr/nwaf441)
Supplement: nwaf441_Supplemental_Files [file nwaf441_supplemental_files.zip › Supplementary_methods.pdf]

Sample husbandry for cross mapping

The grey line of tea geometrid was collected in Yizheng, Yangzhou, China (119°12'E, 32°18'N), while the melanic line was collected in Shengzhou, Shaoxing, China (120°34'E, 29°16'N). Tea geometrids were reared in the laboratory under the conditions of 25 ± 1°C, 75-80% humidity, and 13:11 light/dark photoperiod. Larvae were fed with fresh tea leaves, while adults were provided with honey water for survive. These two lines were maintained in Tea Research Institute, Chinese Academy of Agricultural Science, for screening homozygous offspring for five years (~40-45 generations). Due to the dominant property of the melanic morph, a single male and a single female were selected for mating until the offspring showed fixed body color, and this line was used as a homozygous line for this study.

For backcross mapping (Figure 2A), males and females of both color morphs, at 24 hours post-emergence, were placed into an airtight, transparent cup and mated in darkness. Three female and five male F1 offspring, the hybrid of homozygous melanic and grey parents, were randomly selected and subjected to mating with grey adults. To maximize the population size, a single F1 male was allowed to mate with two or three grey females, while a single F1 female was only paired with one grey male. The body color of offspring was individually observed to determine the phenotype. Melanic and grey offspring were separated after emergence, excluding adults with uncertain traits (approximately <5%).

Genome sequencing of the backcross mapping population

Whole-body genomic DNA of each involved individual (i.e., clearly defined as melanic or grey) was independently prepared using isopropanol. In brief, tissues was homogenized with steel beads in CTAB extraction buffer, followed by digestion with proteinase K at 65°C for 2 hours. After centrifugation, the supernatant was subjected to phenol-chloroform-isoamyl alcohol extraction and isopropanol precipitation. DNA pellet was washed with 75% ethanol and treated with RNase to remove RNA contamination. Additional procedures of phenol-chloroform extraction and isopropanol precipitation were applied to purify the sample, and then ethanol was used to wash DNA. DNA samples with a concentration of less than 100 ng/μL were excluded. As a result, a total of 457 melanic and 447 grey offspring were used for cross-mapping. A bulked segregation analysis (BSA) approach was applied to analyze the genotype-phenotype association. These individual DNA samples were randomly grouped and equally pooled into 20 mixed samples based on sex and body color, i.e., five melanic female pools, five melanic male pools, five grey female pools, and five grey male pools. Each individual sample was diluted to 200 ng/μL using Qubit® 2.0 for pooling. A standard Illumina genomic sequencing library with 500-bp insert was constructed for each mixed sample, according to the standard protocol of DNA Library Prep Kit for Illumina®. Each library was subject for sequencing on an Illumina HiSeq platform under the paired-end mode for at least 50 X sequencing coverage. Both library construction and sequencing were performed by Berry Genomics Co., Ltd., Beijing, China.

BSA-based mapping

Raw reads were filtered and trimmed to remove low-quality bases using seqtk (github.com/lh3/seqtk). Clean reads were aligned to the reference genome of *E. grisescens* [1], using bowtie v2.3.4 [2] with default parameters. Raw mapping results were sorted based on coordinates using SAMTOOLS v0.1.18

[3] and removed with PCR duplicates using Picard v1.82 (broadinsti-tute.github.io/picard/). Variants were called using GATK v3.1.160 [4] across all samples. Bi-allelic SNPs were reserved for subsequent analyses after the following filtering steps: (i) a minor allele frequency (MAF) higher than 2.5%, (ii) a missing ratio lower than 10%, (iii) quality higher than 50, (iv) mapping quality higher than 40, (v) coverage higher than the one-third of the mean value and lower than the two-fold of the mean value, and (vi) showing different genotypes between the parents of the both color morphs.

Allele frequencies of the melanic and grey forms were calculated for each of 10,982,842 filtered SNPs based on the read counts of each morph across samples. According to the expected genetic pattern in BC_1, loci with a fixed derived allele in grey samples and a half ratio of derived allele (0.45 - 0.55) in melanic samples were determined as candidates. Molecular markers were developed within the candidate region for detecting cross events using PCR (Table S5).

To identify structural variations, DNA from a single adult of the grey morph was subject to whole-genome sequencing using the long-read sequencing platform. A 20-Kb insert library was constructed and sequenced on PacBio® RS II Sequencing System by Berry Genomics Co., Ltd. The candidate region of the grey morph was *de novo* assembled by 70 Gb long reads using NextDenovo v2.2-beta.0 [5]. Local synteny within the candidate region between the genomes of both color morphs was evaluated and visualized by progressiveMauve [6] with default parameters. To identify LD blocks, a subset of 542,669 bi-allelic SNPs (MAF < 5% and with presence in all samples) were provided for LDBlockShow [7] with the following parameters: merge minimum SNP number = 5 and Blocktype = 2. The focal genomic region with top association signatures was subject to a manual annotation of genes by TBLASTN of representative lepidopteran homologues downloaded from NCBI.

K-mer-based mapping

To investigate the segregating pattern of structural variations, a *k*-mer-based approach was applied following the method described previously [8]. In brief, 31-mer sequences were extracted from raw sequencing reads of each mixed sample to build *k*-mer presence/absence table using the pipelines of kmersGWAS [8]. The association test between k-mer presence and phenotypes (melanic or grey) was performed using Plink v1.9 [9] with default parameters. K-mers with Bonferroni-corrected $P < 0.01$ were output for further analysis. The subset of K-mers present in all melanic samples but absent in all grey samples were identified as the most significant candidate. Raw reads containing these k-mers were retrieved from the melanic sequencing data, and then mapped to the reference genome, using bowtie v2.3.4, to locate the genomic positions.

Population genetic analysis of wild populations

Wild populations were sampled from five main producing areas of tea in China where grey and melanic morphs simultaneously emerged, including Xiangshan, Shangyu, Longyou, Lin'an, and Tongren (Table S6). Each population was randomly sampled eight melanic and eight grey adults for whole-genome sequencing. Genomic DNA was isolated from each individual as described above. Each sample was subject to a construct paired-end DNA library with a 500 bp insert and sequenced on Illumina HiSeq or Sequel platforms by Berry Genomics Co., Ltd, following the standard approach. Each sample was

sequenced independently for at least 15X coverage. The procedures of SNP calling and filtering were performed as described above.

Principle component analyses based on genome-wide SNPs or those from the candidate region were performed using EIGENSOFT v5.0.2 [10] with default settings. Population structure of wild populations was analyzed using sNMF v1.2 [11] with 10 repetitions for each presumed ancestor number (K). For each run, a subset of 20,000 random SNPs was chosen to initialize this algorithm, and the cross-entropy criterion was simultaneously calculated. Genome-wide association analysis was performed using EMMAX v07Mar2010 [12]. To do this, a BN kinship matrix was first constructed by EMMAX, and then the significance was calculated for each filtered SNP, and subsequently subjected to Bonferroni correction. Outliers without continuous signatures from neighboring SNPs or entirely located in repeats were excluded. K-mer-based approach was also performed separately in each of these wild populations, following the approach as described above. Population statistics of Tajimas' D were calculated using Vcftools v0.1.16 [13] for sliding 10-kb windows. Nucleotide diversity (π) and Patterson's D (ABBA-BABA test) were calculated using the scripts of genomics_general (github.com/simonhmartin). π was calculated for 20-Kb windows with 5-Kb step length. Patterson's D statistics were implemented across 500-Kb windows of Chr. 17, with 100-Kb step length. To measure the intra-populational divergence, the absolute divergence (d_{XY}) was calculated between pairs of individuals within morphs as $d_{XY} = \sum_{ij} x_i y_j d_{ij}$. Betascan [14] was applied to calculate the Beta1* score with sliding window size as 2000 and p parameter as 50.

Annotation of non-coding RNAs in the candidate region

The fifth-instar larval wing-discs and the third day of the pupal wing tissues of both melanic and grey morphs were subjected to transcriptome sequencing. RNA of each sample was independently prepared for three replicates as described above. A standard RNA-seq library was constructed for each replicate of each sample and independently sequenced under paired-end mode on Illumina X Ten. Each library was sequenced for at least 7 Gb raw data. Library construction and sequencing were conducted by Berry Genomics Co., Ltd, following the manufacturer's instructions. The transcriptome data of each sample were independently mapped to the reference genome using HISAT2 v2.1.0 [15] and processed with StringTie v2.0 [16] to predict transcripts. To identify candidate non-coding RNAs or other unannotated genes, the transcriptome data were also merged across samples and subjected to *de novo* assembly using Trinity v2.15.1 [17] with default parameters. The assembled transcripts were estimated with abundance as TPM using Salmon v0.14.1 [18] and mapped to the reference genome using minimap2 [19] with the parameters "-ax splice -uf --secondary=no -C5". Multiple alignment was performed using MAFFT v7.407 [20] and visualized by Jalview v2.11.3.3 [21].

Genomic comparisons between species

To compare the interspecific variations, a *de novo* assembled genome of a closely related species, *E. obliqua,* was aligned to the reference genome of *E. grisescens* using Unimap v0.1-r46-dirty (github.com/lh3/unimap) with parameters "-x asm20 and -r 60k". SyRI v1.6.3 [22] was used to identify intrachromosomal inversions with default parameters, and the resulting alignments were visualized

using Plotsr v1.1.5 [23]. Window-based identity was calculated by custom scripts for 500-Kb windows across Chr. 17 with 5-Kb steps, and 5-Kb windows across the 'melanism locus' with 500-bp steps.

Expression analysis of candidate causal genes

The expression profiles of target genes were analyzed using real-time quantitative PCR (RT-qPCR). Initially, wing discs of the specific developmental stages were dissected and immediately placed into Trizol, then frozen in liquid nitrogen and stored at -80 °C. All wing discs were sampled from female individuals to avoid potential sex bias. Total RNAs were extracted by MolPure® Cell/Tissue miRNA Kit (19331ES50, Yeasen Biotechnology (Shanghai) Co., Ltd., China). Small RNAs were extracted by miRcute miRNA Isolation Kit (DP501, TIANGEN BIOTECH (BEIJING) CO., LTD., China). The messenger RNA (mRNA) of coding genes and '*ivory* transcript' were converted to cDNA using the ReverTra Ace™ qPCR RT Master Mix with gDNA Remover Kit (Toyobo (Shanghai) Biotech CO., LTD., China). For miRNAs, cDNAs were reverse-transcribed using miRNAssayTM qPCR RT Master Mix (MIR-101T, Toyobo (Shanghai) Biotech CO., LTD., China), together with specific stem-loop (SL) primers for each miRNA. All primers were listed in Table S9. RT-qPCR was performed using SYBR® Green Realtime PCR Master Mix (Toyobo (Shanghai) Biotech CO., LTD., China), subjected to the Thermofisher QuantStudio 3 Real-Time PCR System. Ribosomal protein S3a (*EgRps3a*) was used as the reference gene for protein-coding genes and '*ivory* transcript' in the tea geometrid and Ribosomal protein 49 (*BmRp49*) for *cortex* in the silkworm. For *mir-193*, the small nuclear RNA (snRNA) U6 was used as a control. Each gene was assayed for three biological replicates, and the expression profiles were calculated using the $2^{-\Delta\Delta Ct}$ method.

Mutagenesis of focal genes in the tea geometrid

A CRISPR/Cas9-based gene mutagenesis approach was applied to knockout target genes in the tea geometrid. Full-lengths of cortex, parn, 'ivory transcript', and mir-193 in the tea geometrid were manually curated and subjected to PCR verification. Pupal cDNAs were synthesized using the ReverTra Ace qPCR RT Kit (Toyobo (Shanghai) Biotech CO., LTD., China). Target genes were amplified and sequenced to identify potential variations. Specific target sites were designed on candidate genes following the general rule of 5′-(20N)NGG-3′. The single-guide RNA (sgRNA) and Cas9 mRNA were synthesized in vitro using a T7 High Yield RNA Transcription Kit (Ambion). The primers involved are listed in Table S10. Newly laid eggs were fixed on glass slides with glue and injected within 6 hours after oviposition, following the approach described previously [24]. Each egg was injected with 5 μL mixture (300 ng/μL of Cas9 mRNA and 300 ng/μL sgRNA) and incubated for ~7 days until hatching (25°C and 70% relative humidity). A subset of newly hatched larvae was sampled for genotyping to evaluate the overall mutation efficiency. Genomic DNA was prepared from the legs of adult specimens using an Animal Tissue Direct PCR Kit (10184ES50, Yeasen Biotechnology (Shanghai) Co., Ltd., China) for mutation detection by Sanger sequencing.

Gene mutagenesis and overexpression of *cortex* in the silkworm

A non-diapause strain (Nistari) was used in the experiment. Silkworms were fed fresh mulberry leaves and reared under conditions of 25 ± 1°C, 75-80% humidity, and a 13:11 light/dark photoperiod. A

CRISPR/Cas9 system was used for the knockout of *cortex*. The sgRNA was recombined into the piggyBac vector using Hieff Clone® Plus Multi One Step Cloning Kit (10184ES50, Yeasen Biotechnology (Shanghai) Co., Ltd., China). The transgenic strain nos-Cas9 was maintained in the laboratory. The primers involved are listed in Table S10. Newly laid eggs were collected and injected with a mixture of transgenic plasmids (400 ng/µL), helper plasmids (400 ng/µL), and piggyBac transposon mRNAs (200 ng/µL). Injected eggs were incubated for approximately 9 to 10 days until they hatched. Survival G_0 adults were paired for mating to produce G_1 offspring based on the fluorescent signatures. The sgRNA line was crossed with the Cas9 line to generate G2 mutants, which were screened based on the fluorescence markers of EGFP and DsRed.

The Gal4/UAS system was used for over-expression of *cortex* in *B. mori*. The whole coding sequence of *cortex* was inserted into the piggyBac vector as described above. The procedures of micro-injection and mutant screening were performed as described above. The positive UAS-*cortex* offspring was crossed with the GAL4 transgenic silkworm line to produce G2 mutants. Gene expression of *cortex* in mutants was evaluated using RT-qPCR.

Field investigation

Field traps were set in 14 geographic sites from main tea-producing areas in China since 2019. To do this, sticky traps with sex pheromone lure were placed in the tea garden before nightfall, and caught moths were determined with speciation and phenotypes were documented the next morning. To test the effect of temperature and precipitation on fitness, local temperature data were collected from the public database (www.tianqihoubao.com), and local precipitation was collected from the daily gridded precipitation dataset from National Tibetan Plateau/Third Pole Environment Data Center (http://data.tpdc.ac.cn). The developmental threshold temperature was determined according to a previous study [25]. Cumulative temperature (the cumulative daily temperature exceeding developmental threshold temperature at life cycle) and precipitation within a whole generation (~33 days, including ~8 days for the embryonic stage, ~16 days for the larval stage, ~7 days for the pupal stage, and ~2 days for the adult stage) was calculated for each population. Over-wintering populations (i.e., the first generation of each year at each site) were excluded from the analysis. The relationship between cumulative temperature and the proportion of melanic morphs was analyzed through a generalized linear model (GLM) in R with quasibinomial distribution. To compare the difference among populations across consecutive seasons (spring, populations collected before June but excluding over-wintering populations; summer, populations from June to August; autumn, populations from September to the end of the year; winter, over-wintering populations), cumulative temperature and precipitation of the pupal stage (~7days) were used for the correlation analysis and non-parametric ANOVA (Shapiro-Wilk normality test, $W = 0.88$, $P = 1.63\text{x}10^{-5}$).

Fecundity assay

The reproductive capacity was compared between the color morphs of tea geometrids. Five males and three females were placed in a colorless plastic cup for at least 48 hours for mating. Moths that paired up for more than 4 hours were determined as successful copulations and then transferred into a single cup for fertilization without interruption. A total of 271 mating pairs was used for the experiments, with

109 pairs (48 grey and 61 melanic) being tested at 22°C, 101 pairs (56 grey and 45 melanic) at 25°C, and 51 pairs (26 grey and 25 melanic) at 27°C. Each female was allowed to lay eggs for five days and was observed with the population size of offspring based on the hatched larvae; females producing fewer than 20 offspring were excluded from subsequent analysis. To analyze the effects of temperature and morph on fecundity, GLM analysis was applied with a negative binomial (NB) distribution, which showed lower Akaike Information Criterion value than those of Poisson and Gamma distributions. GLM analysis was conducted by *glm.nb* function and logit link of the R package MASS v7.3-60 [26], in which the grey morph and the temperature factor of 25°C were set as the references. Summary statistics of GLM were generated using the Anova (type III) function from the R package car [27]. After statistically significant effects ($P < 0.05$) detected in the models, Tukey-adjusted pairwise post hoc comparisons were performed between the color morphs within each temperature and among temperatures within each morph, using the emmeans function of the R package emmeans v1.11.1 (rvlenth.github.io/emmeans/).

Supplementary References

1. Pan Y, Fang G, Wang Z et al. Chromosome-level genome reference and genome editing of the tea geometrid. *Mol. Ecol. Resour.* 2021; 21(6): 2034-2049.
2. Langmead B, Salzberg SL. Fast gapped-read alignment with Bowtie 2. *Nat. Methods*. 2012; 9(4): 357-359.
3. Li H, Handsaker B, Wysoker A et al. The sequence alignment/map format and SAMtools. *Bioinformatics*. 2009; 25(16): 2078-2079.
4. Van der Auwera GA, Carneiro MO, Hartl C et al. From FastQ data to high-confidence variant calls: the genome analysis toolkit best practices pipeline. *Curr. Pro. Bioinf.* 2013; 43(1): 11.10. 11-11.10. 33.
5. Hu J, Wang Z, Sun Z et al. NextDenovo: an efficient error correction and accurate assembly tool for noisy long reads. *Genome Biol.* 2024; 25(1): 107.
6. Darling AC, Mau B, Blattner FR et al. Mauve: multiple alignment of conserved genomic sequence with rearrangements. *Genome Res.* 2004; 14(7): 1394-1403.
7. Dong S-S, He W-M, Ji J-J et al. LDBlockShow: a fast and convenient tool for visualizing linkage disequilibrium and haplotype blocks based on variant call format files. *Briefings Bioinf.* 2021; 22(4): bbaa227.
8. Voichek Y, Weigel D. Identifying genetic variants underlying phenotypic variation in plants without complete genomes. *Nat. Genet.* 2020; 52(5): 534-540.
9. Purcell S, Neale B, Todd-Brown K et al. PLINK: a tool set for whole-genome association and population-based linkage analyses. *Am. J. Hum. Genet.* 2007; 81(3): 559-575.
10. Galinsky KJ, Bhatia G, Loh P-R et al. Fast principal-component analysis reveals convergent evolution of ADH1B in Europe and East Asia. *Am. J. Hum. Genet.* 2016; 98(3): 456-472.
11. Frichot E, Mathieu F, Trouillon T et al. Fast and efficient estimation of individual ancestry coefficients. *Genetics.* 2014; 196(4): 973-983.
12. Kang HM, Sul JH, Service SK et al. Variance component model to account for sample structure in genome-wide association studies. *Nat. Genet.* 2010; 42(4): 348-354.
13. Danecek P, Auton A, Abecasis G et al. The variant call format and VCFtools. *Bioinformatics.* 2011; 27(15): 2156-2158.
14. Siewert KM, Voight BF. Detecting long-term balancing selection using allele frequency correlation. *Mol. Biol. Evol.* 2017; 34(11): 2996-3005.
15. Kim D, Paggi JM, Park C et al. Graph-based genome alignment and genotyping with HISAT2 and HISAT-genotype. *Nat. Biotechnol.* 2019; 37(8): 907-915.
16. Pertea M, Pertea GM, Antonescu CM et al. StringTie enables improved reconstruction of a transcriptome from RNA-seq reads. *Nat. Biotechnol.* 2015; 33(3): 290-295.
17. Grabherr MG, Haas BJ, Yassour M et al. Full-length transcriptome assembly from RNA-Seq data without a reference genome. *Nat. Biotechnol.* 2011; 29(7): 644-652.
18. Patro R, Duggal G, Love MI et al. Salmon provides fast and bias-aware quantification of transcript expression. *Nat. Methods.* 2017; 14(4): 417-419.

19. Li H. Minimap2: pairwise alignment for nucleotide sequences. *Bioinformatics.* 2018; 34(18): 3094-3100.
20. Katoh K, Misawa K, Kuma Ki et al. MAFFT: a novel method for rapid multiple sequence alignment based on fast Fourier transform. *Nucleic Acids Res.* 2002; 30(14): 3059-3066.
21. Waterhouse AM, Procter JB, Martin DM et al. Jalview Version 2—a multiple sequence alignment editor and analysis workbench. *Bioinformatics.* 2009; 25(9): 1189-1191.
22. Goel M, Sun H, Jiao W-B et al. SyRI: finding genomic rearrangements and local sequence differences from whole-genome assemblies. *Genome Biol.* 2019; 20: 1-13.
23. Goel M, Schneeberger K. Plotsr: visualizing structural similarities and rearrangements between multiple genomes. *Bioinformatics.* 2022; 38(10): 2922-2926.
24. Wang Y, Li Z, Xu J et al. The CRISPR/Cas system mediates efficient genome engineering in Bombyx mori. *Cell Res.* 2013; 23(12): 1414-1416.
25. Ge C, Yin K, Tang M et al. Developmental threshold temperature and effective accumulated temperature of Ectropis grisescens. *Plant Prot.* 2016; 42(6): 110-112.
26. Venables WN, Ripley BD. *Modern applied statistics with S*. Springer Science & Business Media, 2013.
27. Fox J, Weisberg S. *An R companion to applied regression*. Thousand Oaks, 2019.
