# Supplementary material for: The genetics and evolution of moth melanism in the absence of strong natural selection
Source: Natl Sci Rev. 2025 Oct 23;13(1):nwaf441. doi: 10.1093/nsr/nwaf441 (PMC12796802; doi:10.1093/nsr/nwaf441)
Supplement: nwaf441_Supplemental_Files [file nwaf441_supplemental_files.zip › Supplementary_tables.pdf]

Table S1 Phenotypes of various cross-mapping between the grey and melanic morphs of tea geometrids.

Parents[1]	#Offspring	Grey♂	Grey♀	Melanic♂	Melanic ♀	Grey:Melanic
P(Melanic)♀ × P(Grey)♂	125 (F_1)	0	0	60	60	0:120
P(Grey)♀ × P(Melanic)♂	79 (F_1)	0	0	41	38	0:79
F_1♀ × P(Grey)♂	202	50	49	52	51	0.96:1
P(Grey)♀ × F_1♂	111	31	23	26	31	0.95:1
F_1♀× F_1♂	197	23	28	70	76	0.35:1
F_1♂× F_1♀	102	13	16	38	35	0.40:1

[1]P, parent; F_1, the hybrid of grey and melanic parents, which are all melanic.

Table S2 Phenotypic statistics of various backcross mapping progenies.

Parents[1]	♀:♂	#Offspring	Grey♂	Grey♀	Melanic♂	Melanic♀	Grey:Melanic
F_1♀×P(Grey)♂	1:1	123	28	30	32	33	0.89:1
F_1♀×P(Grey)♂	1:1	142	30	36	37	39	0.871
F_1♀×P(Grey)♂	1:1	106	22	32	23	29	1.04:1
P(Grey)♀× F_1♂	2:1	111	31	23	26	31	0.95:1
P(Grey)♀× F_1♂	3:1	79	21	23	15	20	1.26:1
P(Grey)♀× F_1♂	2:1	202	50	49	52	51	0.96:1
P(Grey)♀× F_1♂	3:1	74	21	16	20	17	1:1
P(Grey)♀× F_1♂	3:1	67	18	17	12	20	1.09:1

[1]P, parent; F_1 is the hybrid of grey and melanic parents.

Table S3 Phenotype-genotype association analysis for coding variations of melanin synthesis-related genes.

Gene[1]	#Reference alleles[2]		#Alternative alleles		χ^2	P
	Grey	Melanic	Grey	Melanic		
TH	189	275	14	36	0.382128	0.536467
Ddc	304	361	95	138	0.635458	0.42536
ebony	375	408	38	43	0.998751	0.317613
ebony	344	409	35	40	0.998859	0.317587
ebony	216	226	67	90	0.61863	0.431557
ebony	319	367	35	42	0.998596	0.317651
ebony	334	371	22	38	0.466528	0.494589
ebony	338	401	25	42	0.623435	0.429774
ebony	347	401	26	40	0.754	0.385213
ebony	274	297	34	52	0.543136	0.461136
ebony	287	315	34	52	0.571755	0.449562
ebony	269	312	30	46	0.738003	0.390301
ebony	290	372	21	30	0.987575	0.320336
ebony	289	362	21	25	0.998792	0.317603
ebony	283	361	21	29	0.99505	0.318511
ebony	244	335	91	100	0.62167	0.430427
ebony	312	405	31	32	0.858654	0.354116
ebony	343	421	29	36	0.999978	0.317316
ebony	234	300	143	165	0.910589	0.339958
ebony	233	271	141	118	0.202306	0.652866
ebony	238	253	135	118	0.660484	0.416389
ebony	267	312	101	91	0.487261	0.485152
ebony	249	308	105	93	0.254345	0.614032
ebony	0	2	285	317	0.616509	0.432347
ebony	170	228	89	77	0.132478	0.715877
ebony	203	218	54	88	0.217313	0.641095
aaNAT	131	182	100	118	0.838845	0.359727
aaNAT	116	167	95	114	0.806443	0.369174
aaNAT	83	120	69	75	0.638873	0.424119
aaNAT	78	118	67	72	0.504792	0.477402
aaNAT	68	104	64	68	0.487135	0.485208
aaNAT	405	468	9	9	0.992744	0.319073
aaNAT	173	217	223	256	0.936481	0.333184
aaNAT	15	9	392	456	0.478303	0.489192
aaNAT	23	12	375	419	0.204482	0.651127
aaNAT	186	180	217	262	0.469659	0.493144
aaNAT	193	237	164	165	0.605437	0.436511
aaNAT	10	15	332	434	0.990589	0.319599

[1] No effective SNPs detected within the coding regions of *tan*, *Ppo*, and *yellow*.

[2] Reference and alternative alleles are determined by mapping to the reference genome of the tea geometrid (the melanic morph).

Table S4 Statistics of sequencing data for bulked segregation analysis.

Sample[1]	#Pooled individuals	Total reads	Clean data (Gb)	Average depth (×)
GF-1	41	217,875,506	32.68	42.42
GF-2	41	204,303,948	30.65	39.78
GF-3	43	173,157,970	25.97	33.72
GF-4	45	207,728,142	31.16	40.45
GF-5	50	521,822,312	78.27	101.61
GM-1	48	170,688,146	25.60	33.24
GM-2	40	145,747,994	21.86	28.38
GM-3	41	149,590,686	22.44	29.13
GM-4	48	168,067,972	25.21	32.73
GM-5	49	419,083,484	62.86	81.60
BF-1	40	232,463,692	34.87	45.27
BF-2	41	233,113,500	34.97	45.39
BF-3	41	182,500,956	27.38	35.54
BF-4	43	201,058,444	30.16	39.15
BF-5	52	466,026,244	69.90	90.74
BM-1	45	242,653,500	36.40	47.25
BM-2	46	234,198,106	35.13	45.60
BM-3	48	211,631,244	31.74	41.21
BM-4	49	223,037,638	33.46	43.43
BM-5	51	505,420,254	75.81	98.42

[1] B, melanic; G, grey; F, female; M, male.

Table S5 Molecular markers for detecting crossing events within the candidate region.

Target region on Chr.17	Primer sequence (5'-3')	#Exchanges
3,721,275-3,722,678	F: TTATGAACGAGTCGCGCAGTG<br>R: TCAAGAAATGCGCCTGTACTTGAAG	10/898
4,039,597-4,039,887	F: CTGTCCAACGTGGTGTCACAAG<br>R: CCTAGTACTCGTAGGCACAACCC	0/898
4,058,766-4,059,807	F: CGGCAAAACGTCAATTAAACTAACG<br>R: CAGGAGTTACTTGGTTTGTGTCTC	0/208
4,077,194-4,078,698	F: GTTAGATAGGTCACGACAACTTTC<br>R: CTTAGGATAGAGGCGATAGCTATG	0/208
4,164,868-4,167,409	F: GCTGAACGACCGCTAGTAGAT<br>R: ATAGACGTTAAATCAATTACATGGA	0/898
4,194,341-4,194,694	F: GTGACTAATAAGTCAGCGCCATGAC<br>R: CAGTATTGTGCAAACATTCACCCG	0/898
4,212,141-4,213,158	F: GTGACACACGGGATTAGTGGGTAAC<br>R: AAATCACAAATTTGCGGTATCCGAG	0/898
4,259,571-4,259,866	F: CATCGTCTAGGAATGTGGGTCCTGT<br>R: ATGAGCTCTTTTGATTGAGCTCGAAC	0/898
4,429,570-4,432,086	F: GGACGTTCTTGTGTCGTGTTGA<br>R: CTTGTTGACTCTTGGATCCTGATGA	0/898
4,469,033-4,471,482	F: TCCTGAAAACATGGAGGTGCTAAGA<br>R: ACTATGACCTCATCTTTGAAGTGCC	0/898
4,533,177-4,534,020	F: GAGTCTCCGAGTGTAGCTGTTAAG<br>R: GATCGTCTTTTAGTTCATGGTTGGG	6/631
4,640,378-4,639,843	F: CTGGACTAGTTTCGCCAAGGAGATCA<br>R: CAAGCTCTCGTCGTAGCTGAAGC	6/631

Table S6 Statistics of whole-genome sequencing data of natural populations of tea geometrids.

Population	Location	#Individuals	Clean data (Gb)	Average depth (X)
Xiangshan (Grey)	121°51′E, 29°27′N	8	10.27 – 25.67	13.05 – 32.62
Xiangshan (Melanic)	121°51′E, 29°27′N	8	11.29 – 28.89	14.34 – 36.71
Shangyu (Grey)	120°50′E, 29°48′N	8	14.42 – 35.26	18.32 – 44.81
Shangyu (Melanic)	120°50′E, 29°48′N	8	14.36 – 23.48	18.24 – 29.83
Longyou (Grey)	119°16′E, 29°06′N	8	12.95 – 23.78	16.46 – 30.22
Longyou (Melanic)	119°16′E, 29°06′N	8	13.65 – 21.72	17.34 – 27.59
Lin’an (Grey)	119°35E, 30°16′N	8	13.42 – 19.15	17.05 – 24.33
Lin’an (Melanic)	119°35′E, 30°16′N	8	14.10 – 19.94	17.92 – 25.33
Tongren (Grey)	109°09′E, 27°40′N	8	14.36 – 30.68	18.25 – 38.99
Tongren (Melanic)	109°09′E, 27°40′N	8	13.94 – 24.90	17.71 – 31.65

Table S7 De novo assembled transcripts within the 'melanism locus'.

Assembled ID	Length	L-G	L-B	P-G	P-B	
TRINITY_DN52842_c0_g1_i2	1567					(*cortex*)
TRINITY_DN134839_c0_g1_i1	437					(*ivory*?)
TRINITY_DN103510_c0_g1_i1	268					
TRINITY_DN134020_c0_g1_i1	410					
TRINITY_DN153319_c0_g1_i1	546					
TRINITY_DN158978_c0_g1_i1	873					
TRINITY_DN118032_c0_g1_i1	282					
TRINITY_DN107718_c0_g1_i1	239					
TRINITY_DN42058_c0_g1_i1	2156					(*parn*)
TRINITY_DN59508_c2_g1_i1	705					
TRINITY_DN18427_c0_g1_i4	2346					
TRINITY_DN528_c12_g1_i1	529					
TRINITY_DN137362_c0_g1_i1	539					
TRINITY_DN5975_c5_g2_i1	416					
TRINITY_DN34655_c4_g1_i1	870					
TRINITY_DN87021_c1_g1_i1	863					
TRINITY_DN22493_c3_g1_i1	869					
TRINITY_DN42058_c1_g1_i2	2371					
TRINITY_DN27960_c1_g1_i1	865					
TRINITY_DN34410_c0_g1_i10	3459					
TRINITY_DN34565_c0_g1_i5	2131					
TRINITY_DN19304_c1_g1_i2	841					
TRINITY_DN53219_c0_g1_i1	598					
TRINITY_DN178769_c0_g1_i2	507					
TRINITY_DN81044_c0_g1_i1	390					
TRINITY_DN6657_c0_g1_i10	2637					
TRINITY_DN19893_c0_g1_i1	344					
TRINITY_DN27161_c0_g1_i1	318					
TRINITY_DN11692_c0_g1_i1	218					
TRINITY_DN24524_c0_g1_i1	273					

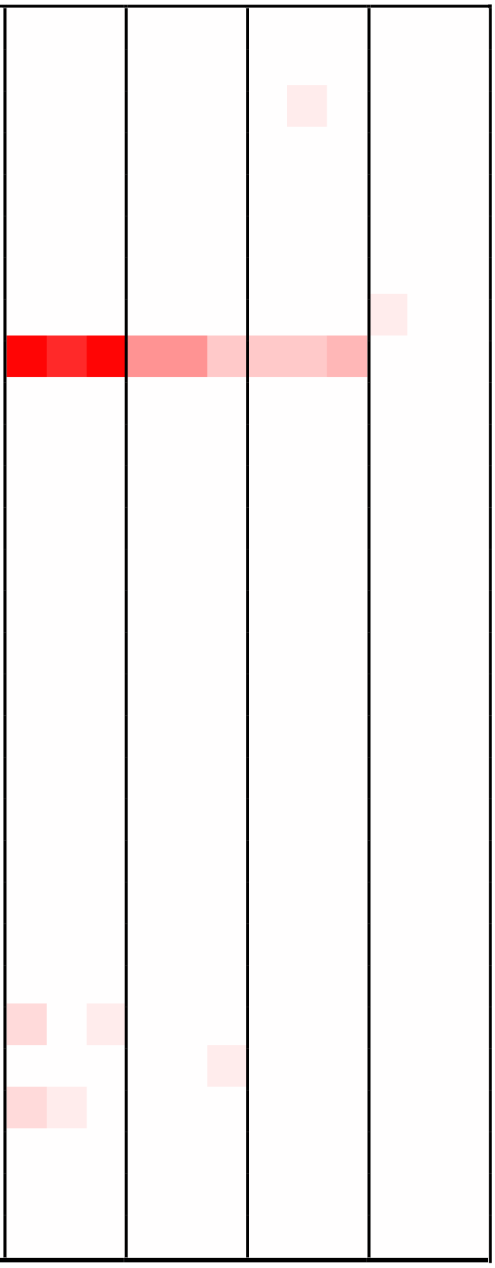

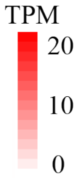

The heatmap shows the abundance of each transcript in three replicates of RNAseq of wing tissues of L-G (5th instar larvae of the grey morph), L-B (5th instar larvae of the melanic morph), P-G (day 3 pupae of the grey morph), and P-B (day 3 pupae of the melanic morph).

Table S8 Detailed information of field investigation.

ID	Location	Time	#Grey	#Melanic	Cumulative temperature (°C)
CD[1]	Chengdu (103°23′E, 30°12′N)	2020/5	293	18	437
TR[1]	Tongren (109°10′E, 27°41′N)	2019/7	155	73	516
XY[1]	Xinyang (113°54′E, 32°04′N)	2020/5	147	2	456
CS	Changsha (113°21′E, 28°28′N)	2020/9	21	3	556
GA[1]	Gao'an (115°7′E, 28°14′N)	2019/8	135	42	703
HS	Huangshan (118°19'E, 29°42'N)	2019/8	64	2	628
JR	Jurong (119°10'E, 31°57'N)	2020/9	80	4	647
LA	Lin'an (119°36′E, 30°17′N)	2019/5	195	12	333
YS	Yushan (120°6'E, 30°4'N)	2020/6	92	9	455
FY	Fuyang (119°51'E, 30°4'N)	2020/8	70	36	676
SX[1]	Shaoxing (120°35'E, 29°59'N)	2020/8	31	7	684
XC[1]	Xinchang (120°54'E, 29°30'N)	2020/9	51	7	635
JH[1]	Jinhua (119°49'E, 28°54'N)	2019/8	393	169	670
XS[1]	Xiangshan (121°51′E, 29°28′N)	2020/8	151	15	614

[1] Populations continuously documented for analyzing seasonal effects (see Figures S12 and S13).

Table S9 Sequence information of primers used in RT-qPCR of this study.

Primer name	Forward primer (5’-3’)	Reverse primer (5’-3’)
m-cort	AGTGTTTGTGACTTATAACCCATGG	TCGGCTTCATCCTCTTGACTATGAC
Eg-parn	GCCCGCATGATTAGTCAATCG	CTTGAACGGAGGTAAACTTGACATG
Eg-Ddc	AGTGGTGCTATTGCCTGCAT	TGCTGTTCCTTGACACGTGT
Eg-tan	TCAACACACTGAGCCCGTAC	GTACATCTGTCTGCCACCCC
Eg-punch	TCAGCAAACTCGCCAGGATT	TTCTGGACTCCACGCATCAC
Eg-ebony	CTCGGAAACAGAAGCCACCT	GTACATGAGGATCGAGCGCA
Eg-AANAT	GCACGGAGACTTCGTAGGAG	GACCCTCATAAGACCGCGTC
Eg-TH	AGCACCTCTGTACAGCCCTA	GAGGGTCTCTAAGGCCTCCA
Eg-PAH	CTGCAAGGAACACAACCACG	CGAATGGTGGCGGATGTACT
Eg-Ppo	GCGTCAGACATCCGTATCGT	TCGGGAAGTAAGCCTCAGG
Eg-yellow2	CCAAGGCCTTCATCCCAGAG	TTGTCGAGGACCCAGAGTCT
RpS3A	GTCTTCGAGGTCTCTTTAGCTGATC	CTCAGGCTGTCTTTGTTGGTGAAC
Bm-Dhpr	CTTAGAAGCCACCCCAGGAA	GTCAGCGGAGTCCATGTACT
Bm-punch	GAACTGCACCTTCCACCATG	TCGTCCGTGTCTTCATCGAA
Bm-Ppo	GAGTTGCTGTTCGATCGTCC	TTCAGCGTCCTTGGGTAGTT
Bm-Ddc	CGCAACGGTCTCATTCCATT	TCAGGGCAGATGAAAGCAGA
Bm-TH	CCGCTTCGAAACACTGGTAG	TCCCATCTTCAACGGTTGGA
Bm-PAH	TTCCGGAAGTTGACAGAGCT	GCAAGTCCAGCCAGAAAGTC
Bm-aaNAT	AGCGTCTACACACTCGTCTT	TGTAGACCCTGGCTTCAAGG
Bm-yellow2	CGAAAGGCTTCATTCCCGAG	GCGGTATGGCGAGACAATTT
Bm-yellow	AACTACCCGGACCAGTTCTC	CGGGTAGGGCGTTAATTTCG
Bm-tan	CGTACGATAAGACCCTGGCT	ATGCATCCTCTGTGTGTCCA
Bm-ebony	ATAACCCTCACGCCACTCAA	CCAAACCGTAGCATAGCACC
Bm-RP49	TCAATCGGATCGCTATGACA	ATGACGGGTCTTCTTGTTGG
qpcr_ivory	AAGTGACTCGAGCGTTCTGC	TCTATGTCGGGAATGCGCTC
SL_miR-193-3p	GTCGTATCCAGTGCAGGGTCCGAGGTATTCGCACTGGATACGACGCTTGGG	
SL_U6	GTCGTATCCAGTGCAGGGTCCGAGGTATTCGCACTGGATACGACCACGATTTTGCG	
qpcr_miR-193-3p_F	GTACTGGCCTGCTAAGTC	
qpcr_U6_F	CTGCGCAAGGATGACACG	
qpcr_universal_R	AGTGCAGGGTCCGAGGTATT	

Table S10 Sequence information of primers used in CRISPR/Cas9 experiments of this study.

Primer name	Sequence (5'-3')
eg-parn-sg7	TAATACGACTCACTATAGGCTACGACGCCTACATCACGTTTTAGAGCTAGAAATAGCAA
eg-parn -sg9	TAATACGACTCACTATAGGTAGTCGTCAGGCAGAGGTGTTTTAGAGCTAGAAATAGCAA
eg-cortex-sg2	TAATACGACTCACTATAGGATCGGTTCGTAGTTCCGAGGGGTTTTAGAGCTAGAAATAGCAA
eg-cortex-sg3	TAATACGACTCACTATACCTGACGCCCCTCAAAATCTACCGTTTTAGAGCTAGAAATAGCAA
eg-cortex-sg7	TAATACGACTCACTATACCACAGGACGGGGGAACTACTCCGTTTTAGAGCTAGAAATAGCAA
sg-Bmcor2-F	GGATAGGTTCGTTCTACCCAGTTTTAGAGCTAGAAATAGCAAGTT
sg-Bmcor2-R	TGGGTAGAACGAACCTATCCACTTGTAGAGCACGATATTTTGTAT
eg_mir-193_g1	TAATACGACTCACTATAGACCGCTAAGACCCTTGCCGGTTTTAGAGCTAGAAATAGCAA
eg_mir-193_g2	TAATACGACTCACTATAGTCGGTGTGTGGTTTCTTACGTTTTAGAGCTAGAAATAGCAA
eg_ivory_g1	TAATACGACTCACTATAAGATGTTAGAAATAGTTCTTGTTTTAGAGCTAGAAATAGCAA
eg_ivory_g2	TAATACGACTCACTATATCTATCAGTGCGCGCGCGCTGTTTTAGAGCTAGAAATAGCAA

Table S11 Detailed statistical information for ecological context analyses.

Variables	Estimate	Standard error	*LRχ²/z*	*P*
GLM Result				
Intercept	5.64775	0.09419	59.964	< 2e-16***[1]
Melanic	-0.37732	0.14104	-2.675	0.00747**
22°C	0.02908	0.14184	0.205	0.83755
27°C	-0.18251	0.16769	-1.088	0.27643
Melanic:22°C	-0.09977	0.19971	-0.5	0.61737
Melanic:27°C	0.12075	0.24961	0.484	0.62857
Anova test for GLMs				
Morph			7.0249	0.008039**
Temperature			1.5604	0.458309
Morph:Temperature			0.8072	0.667917
Pairwise post hoc comparisons				
22°C: Grey-Melanic	0.477	0.141	3.374	0.0007***
25°C: Grey-Melanic	0.377	0.141	2.675	0.0075**
27°C: Grey-Melanic	0.257	0.206	1.246	0.2128
Grey: 25°C-22°C	-0.02908	0.142	-0.205	0.9771
Grey: 25°C-27°C	0.18251	0.168	1.088	0.5213
Grey: 22°C-27°C	0.21159	0.175	1.212	0.4462
Melanic: 25°C-22°C	0.07068	0.141	0.503	0.87
Melanic: 25°C-27°C	0.06177	0.185	0.334	0.9403
Melanic: 22°C-27°C	-0.00892	0.179	-0.05	0.9986

[1] Significance: ** $P < 0.01$; *** $P < 0.001$.
